# Hyaluronan network remodeling by ZEB1 and ITIH2 enhances the motility and invasiveness of cancer cells

**DOI:** 10.1172/JCI180570

**Published:** 2025-04-03

**Authors:** Sieun Lee, Jihye Park, Seongran Cho, Eun Ju Kim, Seonyeong Oh, Younseo Lee, Sungsoo Park, Keunsoo Kang, Dong Hoon Shin, Song Yi Ko, Jonathan M. Kurie, Young-Ho Ahn

**Affiliations:** 1 Department of Molecular Medicine and Inflammation-Cancer Microenvironment Research Center, College of Medicine, Ewha Womans University, Seoul, South Korea.; 2 Deargen Inc., Daejeon, South Korea.; 3 Department of Microbiology, College of Science and Technology, Dankook University, Cheonan, South Korea.; 4 Research Institute and Department of Cancer Biomedical Science, Graduate School of Cancer Science and Policy, National Cancer Center, Goyang, South Korea.; 5 UroGyn Efficacy Evaluation Center, Ewha Womans University Mokdong Hospital, Seoul, South Korea.; 6 Department of Thoracic/Head and Neck Medical Oncology, University of Texas MD Anderson Cancer Center, Houston, Texas, USA.

**Keywords:** Cell biology, Oncology, Extracellular matrix, Lung cancer

## Abstract

Hyaluronan (HA) in the extracellular matrix promotes epithelial-mesenchymal transition (EMT) and metastasis; however, the mechanism by which the HA network constructed by cancer cells regulates cancer progression and metastasis in the tumor microenvironment (TME) remains largely unknown. In this study, inter-α-trypsin inhibitor heavy chain 2 (ITIH2), an HA-binding protein, was confirmed to be secreted from mesenchymal-like lung cancer cells when cocultured with cancer-associated fibroblasts. ITIH2 expression is transcriptionally upregulated by the EMT-inducing transcription factor ZEB1, along with HA synthase 2 (HAS2), which positively correlates with ZEB1 expression. Depletion of ITIH2 and HAS2 reduced HA matrix formation and the migration and invasion of lung cancer cells. Furthermore, ZEB1 facilitates alternative splicing and isoform expression of CD44, an HA receptor, and CD44 knockdown suppresses the motility and invasiveness of lung cancer cells. Using a deep learning–based drug-target interaction algorithm, we identified an ITIH2 inhibitor (sincalide) that inhibited HA matrix formation and migration of lung cancer cells, preventing metastatic colonization of lung cancer cells in mouse models. These findings suggest that ZEB1 remodels the HA network in the TME through the regulation of ITIH2, HAS2, and CD44, presenting a strategy for targeting this network to suppress lung cancer progression.

## Abstract 2

ZEB1 establishes a pro-invasive hyaluronan network through the regulation of ITIH2, HAS2, and CD44. An ITIH2 inhibitor (sincalide) prevents lung cancer invasion and progression.

## Graphical abstract

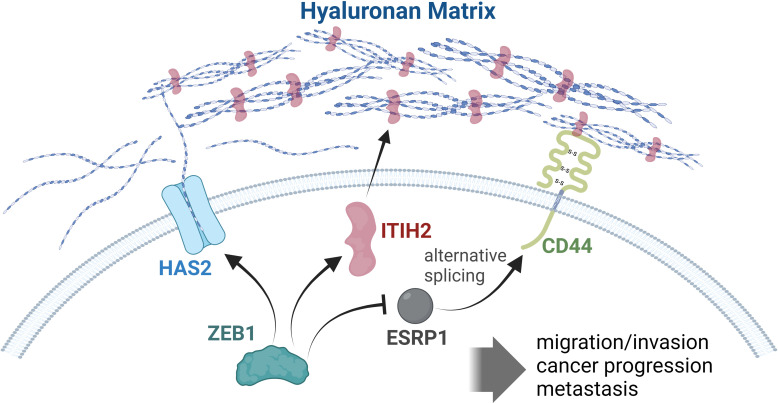

## Introduction

The extracellular matrix (ECM) constitutes a dynamic network of proteins and carbohydrates enveloping cells, offering mechanical support, regulating cell behavior, and influencing tissue architecture (1). Comprising diverse elements, such as collagen, proteoglycans, glycoproteins, and glycosaminoglycans (GAGs), the ECM includes hyaluronan (HA), a pivotal non-sulfated GAG. HA plays a major role in the ECM and is implicated in various physiological and pathological processes, including tissue development, wound healing, inflammation, and cancer (2). Recent research indicates dysregulation of HA and its pathways in cancers, such as breast, pancreatic, and lung cancer (3–5). Elevated HA levels in the tumor microenvironment (TME) correlate with poor prognosis and therapy resistance (6, 7). Understanding the involvement of HA-associated pathways in cancer pathogenesis offers potential avenues for therapeutic strategies in cancer treatment.

The regulation of HA deposition and matrix formation in the ECM involves a complex interplay between enzymes, receptors, and signaling pathways. HA synthases (HAS1, HAS2, and HAS3) govern HA synthesis in the ECM (2). Hyaluronidases (HYAL family) degrade HA, breaking it into smaller fragments that the body can clear (8). HA receptors, such as CD44 and RHAMM, bind to HA, influencing cell adhesion, migration, and proliferation (9–11). Signaling pathways, such as TGF-β and Wnt, along with inflammatory cytokines, can modulate HAS, HYAL, and HA receptor expression, leading to HA matrix remodeling (12–14). HA-binding proteins, including versican and inter-α-trypsin inhibitor heavy chain (ITIH) family members, play crucial roles in regulating HA cable formation in the ECM (15, 16). Versican binds to HA via its G1 domain, interacting with other ECM molecules such as fibronectin and collagen, promoting HA cable assembly by cross-linking HA molecules and enhancing cell adhesion, migration, and proliferation (16). ITIH proteins, binding to HA through their C-terminal domains, interact with other ECM proteins, such as fibronectin and laminin, promoting HA cable formation by stabilizing HA-ITIH complexes and facilitating their incorporation into the ECM (15). These HA-binding proteins play vital roles in regulating the deposition and organization of HA in the ECM.

Epithelial-mesenchymal transition (EMT) induces alterations in cancer cell behavior, such as heightened invasiveness, stemness, and therapy resistance, contributing to tumor progression and metastasis in epithelial cancers (17). EMT influences the production, deposition, and turnover of ECM components, including HA (18, 19). In the course of EMT, cancer cells elevate HAS2 expression, resulting in increased production and deposition of HA in the ECM (19). Furthermore, CD44 isoform switching induced by EMT can modify the binding affinity and downstream signaling of HA, further shaping cancer cell behavior (20). Changes in HA deposition and organization due to EMT can impact the biophysical properties of the tumor, influencing the migratory and invasive capabilities of cancer cells. For instance, augmented HA deposition in the ECM can elevate matrix stiffness, potentially promoting cancer cell invasion by providing a physical scaffold for cell migration (1, 21).

Our prior research revealed that the EMT status of lung cancer cells influences their interaction with cancer-associated fibroblasts (CAFs) in the ECM (22). Through transcriptome and secretome analyses, we identified ITIH2 as a key regulator of CAF motility in mesenchymal-like cancer cells. In this study, we demonstrated that ZEB1 controls ITIH2 and other components involved in the reconstruction of the HA matrix, propelling changes in the lung TME that enhance cancer cell migration and invasion.

## Results

### ZEB1 is a transcription activator of ITIH2, an HA-binding protein.

**Figure 1:**
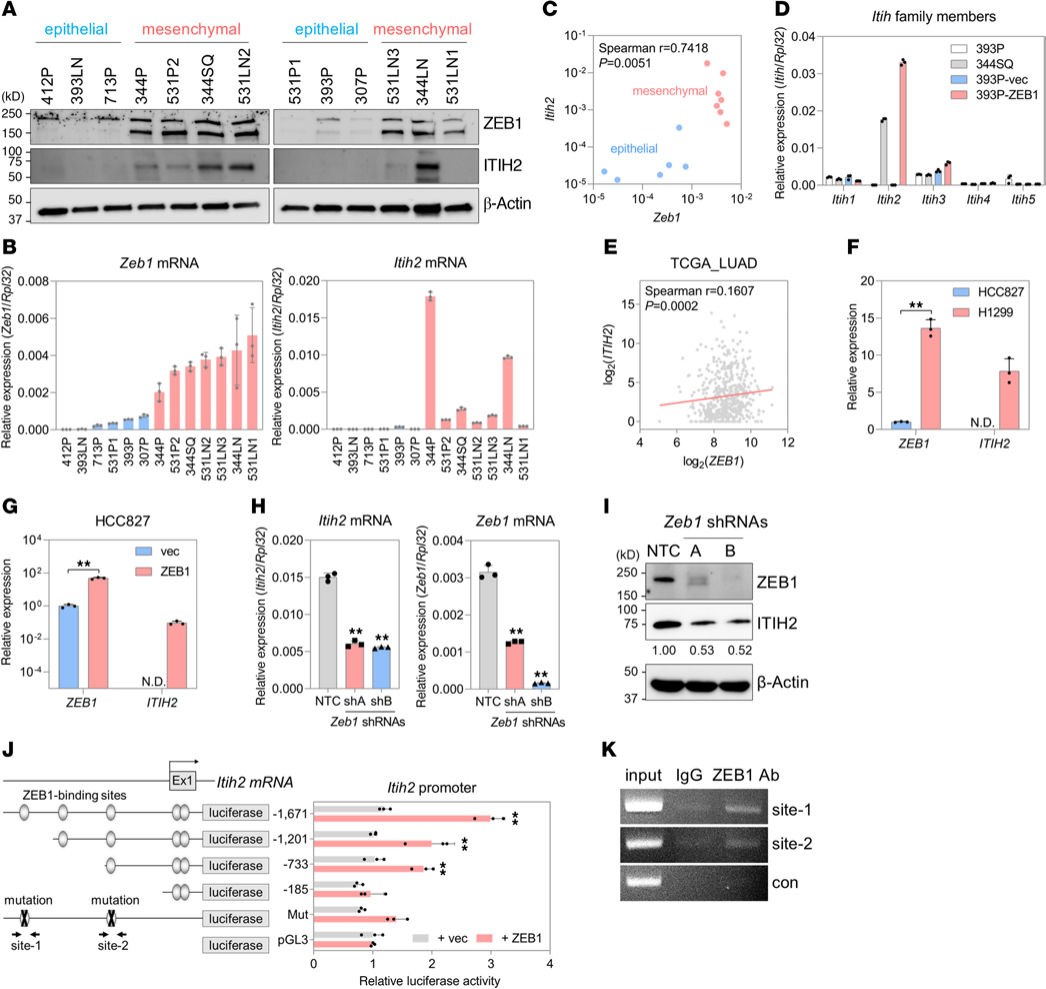
ZEB1 is a transcription factor of ITIH2. (**A**) Western blot showing expression of ZEB1 and ITIH2 in murine lung cancer cells. β-Actin served as a loading control. (**B**) Quantitative reverse transcription PCR (qRT-PCR) analysis of *Zeb1* and *Itih2* mRNA in epithelial-like (blue) and mesenchymal-like (red) murine lung cancer cells. *Rpl32* was used as a reference gene. (**C**) Scatterplot illustrating the correlation between *Zeb1* and *Itih2* mRNA levels in qRT-PCR data of murine lung cancer cells (*n* = 13). (**D**) qRT-PCR of the ITIH family members (*Itih1*–*Itih5*). (**E**) Scatterplot depicting the correlation between *ZEB1* and *ITIH2* mRNA levels in TCGA-LUAD data (*n* = 517). (**F** and **G**) qRT-PCR analysis of *ZEB1* and *ITIH2* mRNA in HCC827 and H1299 cells. Expression levels were normalized to *ZEB1* in HCC827 (**F**) or HCC827-vector (**G**). N.D., not detected. **P < 0.01 by 2-tailed Student’s *t* test. (**H**) qRT-PCR showing *Itih2* and *Zeb1* mRNA levels in 344SQ-ZEB1 knockdown (KD) or control (NTC) cells. **P < 0.01 by 1-way ANOVA followed by Dunnett’s multiple-comparison test. (**I**) Western blot displaying expression of ZEB1 and ITIH2 in 344SQ-ZEB1-KD or control cells. Densitometric analysis (numbers below the ITIH2 blot) is shown relative to NTC (set at 1.0). (**J**) Luciferase reporter assay of *ITIH2* promoter activity. **P < 0.01 by 1-way ANOVA followed by Dunnett’s multiple-comparison test (compared with pGL3+ZEB1). (**K**) ChIP assay on the *ITIH2* promoter region. Cross-linked chromosomal DNA fragments were immunoprecipitated with an anti-ZEB1 antibody or control IgG. Eluted DNA fragments were used as templates for PCR with primer sets covering putative ZEB1-binding sites (site-1 and -2) or with a negative control primer set (con). Data represent the mean ± SD from a single experiment with biological replicates (*n* = 3, unless otherwise specified) and are representative of at least 3 independent experiments.

In a previous study, we established that CAFs induce the secretion of ITIH2, an HA-binding protein, by lung cancer cells (22). Lung cancer cells expressing high levels of ZEB1 secreted more ITIH2 compared with cells with low ZEB1 expression. This observation led us to hypothesize that ZEB1 plays a crucial role in regulating ITIH2 expression in lung cancer cells. To test this hypothesis, we assessed ITIH2 and ZEB1 protein levels in 13 murine lung cancer cell lines derived from *Kras*/*Trp53*-mutant mice (23). Both ITIH2 and ZEB1 protein levels were elevated in mesenchymal-like lung cancer cells compared with their epithelial-like counterparts (Figure 1A). The mRNA levels of *Itih2* and *Zeb1* were markedly higher in mesenchymal-like lung cancer cells than in epithelial-like cells (Figure 1B), with a positive correlation observed between their expression levels in all 13 murine lung cancer cell lines (Figure 1C). Among the 5 ITIH family members examined, only *Itih2* was upregulated in mesenchymal-like lung cancer cells (Figure 1D).

Additionally, analysis of The Cancer Genome Atlas (TCGA) data for lung adenocarcinoma (LUAD) revealed a positive correlation between *ITIH2* and *ZEB1* mRNA levels (Figure 1E). In human lung cancer cells, *ITIH2* and *ZEB1* mRNA levels were substantially higher in mesenchymal-like lung cancer cells (H1299) than in epithelial-like cells (HCC827; Figure 1F) (24). Overexpression of ZEB1 in epithelial-like 393P cells and HCC827 cells upregulated *ITIH2* mRNA expression (Figure 1, D and G), while ZEB1 depletion in 344SQ murine lung cancer cells downregulated ITIH2 expression (Figure 1, H and I). To elucidate the direct relationship between ITIH2 and ZEB1, we conducted *Itih2* promoter luciferase assays, demonstrating that ZEB1 enhanced the promoter activity of *Itih2* (Figure 1J). Deletion or mutation of putative ZEB1-binding sites on the *Itih2* promoter abolished the promoter activation induced by ZEB1 (Figure 1J). Furthermore, chromatin immunoprecipitation (ChIP) assays confirmed the direct binding of ZEB1 to the *Itih2* promoter region (Figure 1K). Analysis of public ZEB1 ChIP-sequencing data in MCF7 breast cancer cells (Gene Expression Omnibus GSE190095) revealed ZEB1 binding to the *ITIH2* promoter region (Supplemental Figure 1; supplemental material available online with this article; https://doi.org/10.1172/JCI180570DS1). These findings provide compelling evidence that ZEB1 functions as a transcriptional activator of ITIH2 in lung cancer cells.

### ITIH2 facilitates the migration and metastatic colonization of lung cancer cells.

**Figure 2:**
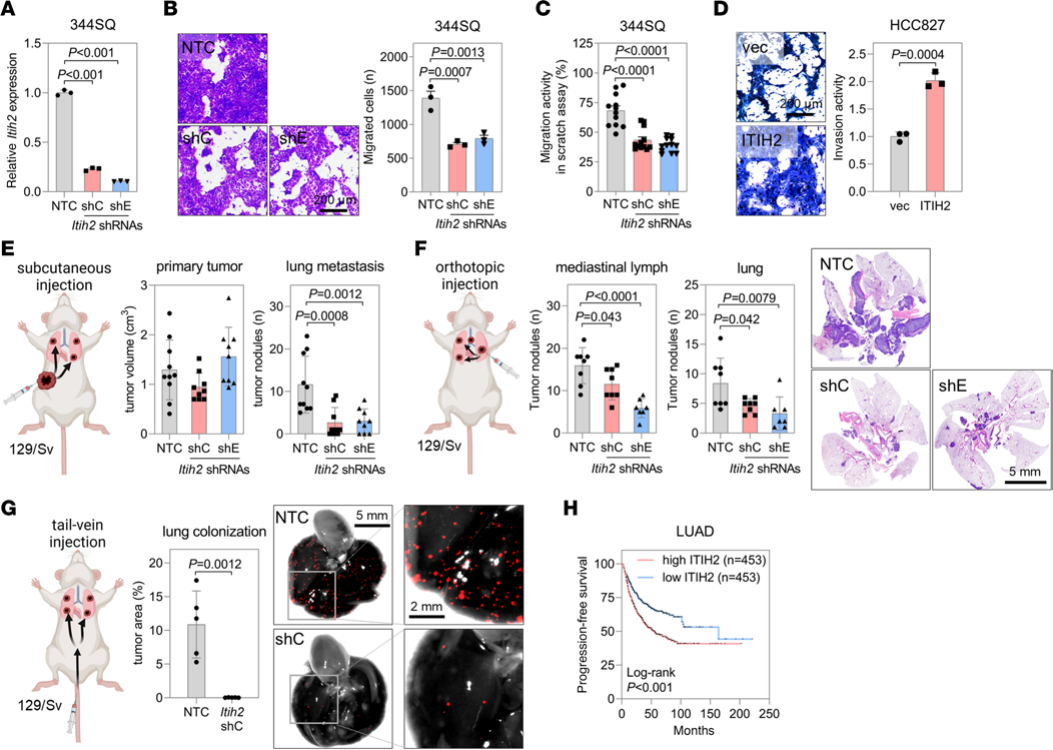
ITIH2 KD inhibits the migration and invasion of lung cancer cells. (**A**) qRT-PCR analysis of *Itih2* mRNA levels in 344SQ cells transduced with *Itih2* shRNAs (shC or shE). *P* values from 1-way ANOVA followed by Dunnett’s multiple-comparison test. (**B**) Boyden chamber migration assay in 344SQ-ITIH2-KD cells. After 24 hours, migrated cells were stained with crystal violet. *P* values determined by 1-way ANOVA followed by Dunnett’s multiple-comparison test. Scale bar: 200 μm. (**C**) Scratch migration assay in 344SQ-ITIH2-KD cells. Migration activity, (1 – [scratch area ratio of 24 hours to 0 hours]) × 100%, was measured using ImageJ (NIH). Mean ± SD (*n* = 12). *P* values determined by 1-way ANOVA followed by Dunnett’s multiple-comparison test. (**D**) Boyden chamber invasion assay in HCC827 cells overexpressing ITIH2. *P* value determined by 2-tailed Student’s *t* test. Scale bar: 200 μm. (**E**) Mouse subcutaneous injection of 344SQ-ITIH2-KD cells. After 6 weeks of injection, the primary tumor volume and lung metastatic tumor nodules were measured at necropsy (*n* = 9 or 10). *P* values determined by 1-way ANOVA followed by Dunnett’s multiple-comparison test. (**F**) Mouse orthotopic injection of 344SQ-ITIH2-KD cells. After a week of injection, the tumor nodules on mediastinal lymph nodes and lungs were measured at necropsy (*n* = 7 or 8). H&E staining results of the lung sections are described. *P* values determined by 1-way ANOVA followed by Dunnett’s multiple-comparison test. Scale bar: 5 mm. (**G**) Mouse tail-vein injection of 344SQ-ITIH2-KD cells. After 10 days of injection, tumor nodules colonized in the lungs were measured at necropsy under a fluorescent microscope (*n* = 5). *P* values determined by 2-tailed Student’s *t* test. Scale bars: 5 mm (left), 2 mm (right). (**H**) Kaplan-Meier curve for progression-free survival in patients with LUAD was generated from the Kaplan-Meier plotter (66). Data represent the mean ± SD from a single experiment with biological replicates (*n* = 3, unless otherwise specified) and are representative of at least 3 independent experiments.

To elucidate the functional role of ITIH2 in lung cancer cells, we used shRNAs to deplete ITIH2 in murine mesenchymal-like lung cancer cells (344SQ, 344LN, and 531LN2) (Figure 2A and Supplemental Figure 2, A, D, and G) (24). ITIH2 knockdown (KD) had minimal impact on cellular growth or the expression of EMT markers (Supplemental Figure 2, B and C). However, subsequent observations revealed a marked inhibition of cell migration and invasion, as demonstrated by Transwell and scratch assays (Figure 2, B and C, and Supplemental Figure 2, E, F, H, and I). Conversely, ectopic expression of ITIH2 in HCC827 and A549 human lung cancer cells (Supplemental Figure 2J) enhanced cell migration and invasion (Figure 2D and Supplemental Figure 2K).

The 344SQ cell line, mesenchymal-like cells with high ZEB1 expression, was used to study lung cancer progression and metastasis in 129/Sv syngeneic mice (23, 24). Following subcutaneous injection, tumors develop at the injection site and metastasize to the lungs within 5–6 weeks. Beyond 6 weeks, metastasis spreads to multiple organs, such as the liver, kidney, and intestines, often necessitating euthanasia. ZEB1 KD or targeting with microRNA-200 in 344SQ cells reduced their metastatic potential, indicating that ZEB1 is a key regulator of metastasis in this system (23, 25, 26). Additionally, ITIH2-depleted 344SQ cells demonstrated impaired metastasis to distant organs such as the lung, liver, kidney, spleen, and brain compared with control cells when injected into syngeneic mice (129/Sv) subcutaneously (Figure 2E and Supplemental Figure 3A). Furthermore, in an orthotopic injection model, ITIH2 depletion suppressed local invasion and lymph node metastases (Figure 2F). Mice that underwent surgery during the orthotopic injection showed minimal signs of systemic inflammation or infection (Supplemental Figure 3B). ITIH2 depletion also suppressed lung colonization in a tail-vein injection model (Figure 2G). Because of rapid disease progression in the orthotopic and tail-vein injection models, mice were euthanized 7–10 days after injection, which was insufficient time to assess distant metastasis. ITIH2 overexpression enhanced the invasive potential of lung cancer cells in mouse orthotopic injection (Supplemental Figure 2L). Moreover, ITIH2 add-back in ZEB1-KD cells restored their motility and local invasive potential (Supplemental Figure 2, M and N). Analysis of publicly available LUAD datasets using the Kaplan-Meier plotter (27) revealed that patients with high *ITIH2* expression exhibited a worse progression-free survival rate than those with low *ITIH2* expression (Figure 2H). In the same database, *ITIH2* expression was higher in lung metastatic tumors compared with primary tumors (Supplemental Figure 2O). ZEB1 has also been reported to be negatively correlated with overall survival in patients with lung cancer (20, 28). In primary tumors, both ZEB1 and ITIH2 were highly expressed at the invasive front of the tumor edge compared with the central region, indicating that they promote invasive potential. Consistent with these findings, high expression of ZEB1 and ITIH2 was observed throughout metastatic tumors (Supplemental Figure 3C). These results strongly indicate that ITIH2, regulated by ZEB1, is indispensable for enhancing the migration and invasion of lung cancer cells, as well as local tumor progression.

### ITIH2’s role in establishing a pro-migratory extracellular environment.

**Figure 3:**
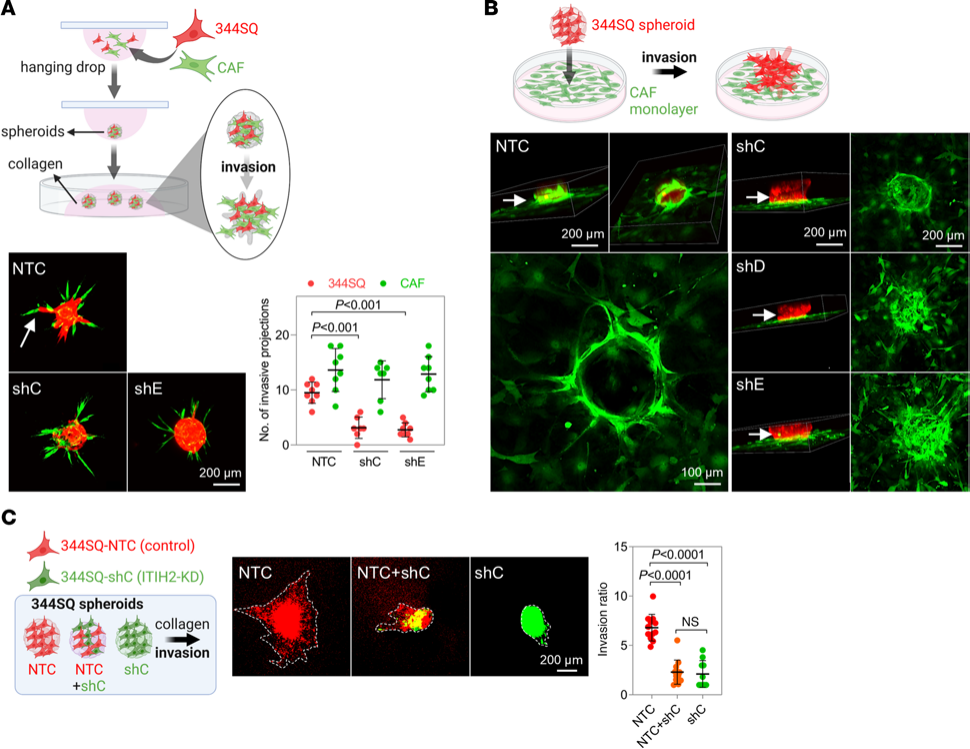
ITIH2 KD inhibits the interaction between cancer cells and CAFs. (**A**) Confocal micrographs of multicellular aggregates containing CAF-led invasive structures (arrows). Spheroids were created with both CAFs (labeled with GFP) and 344SQ-NTC or ITIH2-KD cells (shC and shE, labeled with mCherry). The spheroid invasion ratios were calculated by ImageJ software after 24 hours of incubation. Mean ± SD (NTC, *n* = 8; shC, *n* = 7; shE, *n* = 7). *P* values determined by 1-way ANOVA followed by Dunnett’s multiple-comparison test. Scale bar: 200 μm. (**B**) Confocal micrographs of spheroid overlay cultures where 344SQ cancer cell spheroids (NTC or shC–E, mCherry) were placed on top of a confluent monolayer of CAFs (GFP), which were cultured for 24 hours before the spheroid seeding. Multicellular aggregates were further incubated for another 24 hours to allow migration and invasion. Arrows indicate the lateral surface of cancer cell aggregates interacting with surrounding CAFs. Scale bars: 200 μm (top), 100 μm (bottom left). (**C**) Spheroid invasion assay in 344SQ-ITIH2-shC (green), NTC (red), and mixed NTC and shC (NTC+shC) cells. The cancer spheroids were composed of NTC only, shC only, and a mix of both NTC and shC. To differentiate between the cell types, NTC cells were labeled with red fluorescence and shC cells with green fluorescence. After 48 hours, spheroids were embedded in collagen gels. The spheroids were imaged using a fluorescence microscope (middle panel), and the spheroid invasion ratio was calculated using ImageJ software (right) after 24 hours of incubation. Mean ± SD (NTC, *n* = 12; shC, *n* = 10; NTC+shC, *n* = 12). *P* values determined by 1-way ANOVA followed by Dunnett’s multiple-comparison test. Scale bar: 200 μm. Data represent the mean ± SD from a single experiment with biological replicates and are representative of at least 3 independent experiments.

As ITIH2 promotes cellular motility, invasiveness, and the formation of HA bundles in the ECM, it is proposed that ITIH2 modulates heterotypic cellular interactions within the TME. In a 3D collagen gel–based invasion assay with cocultures of lung cancer cells and CAFs, 344SQ cells invaded following CAFs at the front of invasive projections as described previously (22). However, ITIH2-depleted 344SQ cells failed to invade along the CAFs (Figure 3A). We subsequently performed spheroid overlay cultures by placing 344SQ cancer cell spheroids (red) on a confluent layer of CAFs (green), which had been cultured for 24 hours prior to the spheroid seeding (Figure 3B). The 344SQ spheroids pushed CAFs outward and invaded the feeder layer, with the CAFs climbing up the lateral surface of the 344SQ spheroid as they were pushed outward. Meanwhile, ITIH2 depletion again hindered the 344SQ spheroids from invading the CAF feeder layer. To further validate the impact of the HA matrix facilitated by ITIH2 on cell-cell interaction during invasion into the ECM, we labeled control 344SQ cells (nontargeting control [NTC]) with red fluorescence and ITIH2-depleted cells (shRNA-C [shC]) with green fluorescence. After mixing NTC and shC cells to form spheroids, we embedded these spheroids in collagen gels (Figure 3C). Interestingly, spheroids composed of both NTC and shC cells also exhibited reduced invasive activity, comparable to those consisting solely of shC cells, suggesting that the depletion of the HA matrix due to ITIH2 KD not only impairs the invasive activity of the KD cells themselves but also negatively impacts neighboring cancer cells. These findings underscore ITIH2’s role in creating a pro-migratory extracellular environment for neighboring cells through the construction of the HA network.

### ZEB1’s contribution to HA network formation.

**Figure 4:**
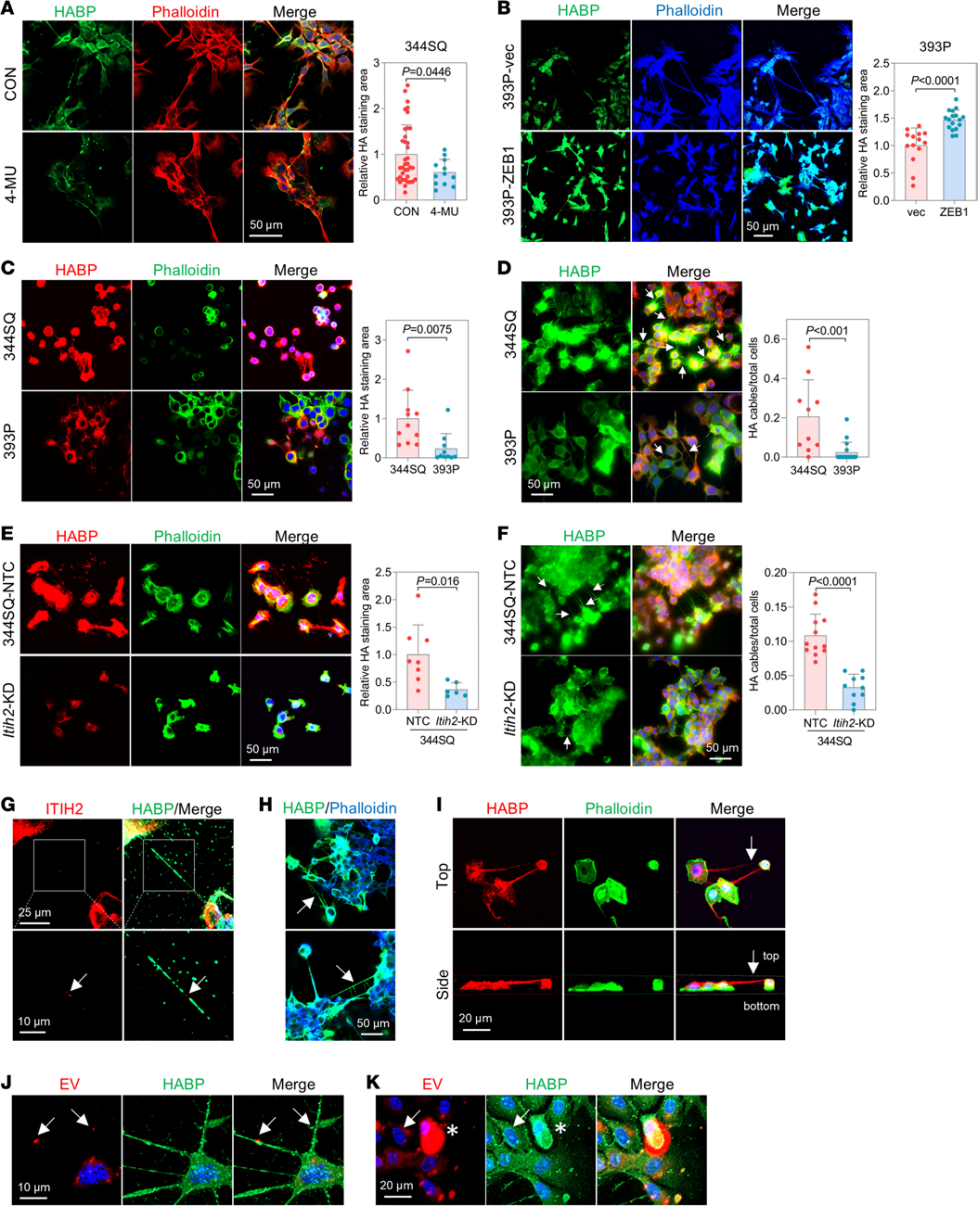
ZEB1 and ITIH2 facilitate the formation of the HA matrix and cables. (**A**) Confocal microscopy of 344SQ cells with or without 4-MU treatment (1 mg/mL for 24 hours) stained with HABP, phalloidin, and DAPI. Mean ± SD (control [CON], *n* = 40; 4-MU, *n* = 12). (**B**) Confocal microscopy of 393P-vec and 393P-ZEB1 cells. Mean ± SD (393P-vec, *n* = 14; 393P-ZEB1, *n* = 17). (**C**) Confocal microscopy of 344SQ and 393P cells. Mean ± SD (344SQ, *n* = 11; 393P, *n* = 10). (**D**) Confocal *Z*-stack images of 344SQ and 393P cells. The arrows indicate HA cables. Mean ± SD (393P, *n* = 17; 344SQ, *n* = 10). (**E**) Confocal microscopy of 344SQ-ITIH2-KD cells. Mean ± SD (344SQ-NTC, *n* = 8; ITIH2-KD, *n* = 6). (**F**) Confocal *Z*-stack images of 344SQ-ITIH2-KD cells. Arrows indicate HA cables. Mean ± SD (344SQ-NTC, *n* = 12; ITIH2-KD, *n* = 10). Scale bars in **A**–**F**: 50 μm. (**G**) Confocal *Z*-stack images of 344SQ cells. The arrows indicate ITIH2 on the HA cable. Scale bars: 25 μm (top), 10 μm (bottom). (**H**) Confocal *Z*-stack images of 344SQ cells. The arrows indicate that HA cables are not colocalized with cytoskeletons. Scale bar: 50 μm. (**I**) Confocal *Z*-stack images of 344SQ cells. The arrows indicate an HA cable forming a bridge between 2 cells. Scale bar: 20 μm. (**J**) Confocal *Z*-stack images of 344SQ cells. RFP-labeled EVs were treated to control 344SQ cells for 24 hours. The arrows indicate the captured EVs on HA cables. Scale bar: 10 μm. (**K**) Confocal *Z*-stack images of the same 344SQ cells as in **J**. The arrows indicate a cell with less HA matrix, and the asterisks indicate a cell with abundant HA matrix. Scale bar: 20 μm. Data represent the mean ± SD from a single experiment with biological replicates and are representative of at least 3 independent experiments. *P* values determined by 2-tailed Student’s *t* test.

ITIH2 actively contributes to the formation of HA-rich extracellular cables by forming covalent bonds with HA, detectable using HA-binding protein (HABP) as a specific probe for HA, indicating the presence of an extracellular HA matrix (15). The abundance of the HA matrix surrounding 344SQ cells was reduced with the inhibition of HA synthesis by 4-methylumbelliferone (4-MU) (29) or hyaluronidase treatment (Figure 4A and Supplemental Figure 4, A–C). Additionally, the formation of the extracellular HA matrix was less prominent and narrower in 393P and HCC827 cells with low levels of ZEB1 and ITIH2 compared with 344SQ and H1299 cells, respectively (Figure 4C and Supplemental Figure 4D). ZEB1 or ITIH2 overexpression in 393P and HCC827 cells increased the formation of the HA matrix (Figure 4B and Supplemental Figure 4, E–G), while ITIH2 KD in 344SQ cells led to a reduction in the extent of the extracellular HA matrix (Figure 4E). Gefitinib-resistant HCC827 cells that had undergone the EMT process (30) and expressed high levels of ZEB1 developed a more extensive HA matrix compared with the control (Supplemental Figure 4, H–J). 4-MU treatment effectively blocked HA synthesis and HA matrix formation even with ectopic ZEB1 overexpression (Supplemental Figure 4, K and L). During the scratch assay, the HA matrix was enriched around the leading edge of migrating cancer cells, implying its role in facilitating cell migration (Supplemental Figure 5A). Forced expression of ZEB1 in 393P cells promoted HA matrix formation, whereas depletion of ZEB1 and ITIH2 in 344SQ cells suppressed HA matrix formation (Supplemental Figure 5, B–D). These observations emphasize the pivotal role of ZEB1 in promoting the development of an extracellular HA environment.

As anticipated, a higher presence of HA cables was evident in 344SQ cells compared with 393P cells or ITIH2-KD cells (Figure 4, D and F). ITIH2 was identified as a component of these cables, as it forms bundles or cables by binding to HA (Figure 4G). These HA cables did not overlap with the actin cytoskeleton (Figure 4H), signifying that they were not extensions of the cellular membrane. These HA cables served as intercellular connections, resembling suspension bridges between cells (Figure 4I), suggesting potential intercellular communication through this structure. HA matrices and cables are recognized for their role in facilitating the release and transport of extracellular vesicles (EVs) (31, 32). Moreover, breast cancer cells produce HA-rich trails that contain EVs, aiding in directional cell migration (33). These findings imply that HA cables are not merely passive structures but rather active extracellular structures involved in regulating cell-to-cell interactions. Consistent with this, EVs were observed bound to HA cables (Figure 4J), and cells with a well-developed HA matrix exhibited increased binding and uptake of EVs (Figure 4K and Supplemental Figure 5E). 344SQ cells enriched with an HA matrix exhibited enhanced EV binding and uptake compared with 393P cells (Supplemental Figure 5F). Although further investigation is warranted, these findings propose that the HA matrix plays a pivotal role in cell migration and facilitates intercellular communication.

### ZEB1 governs the cellular response to HA.

**Figure 5:**
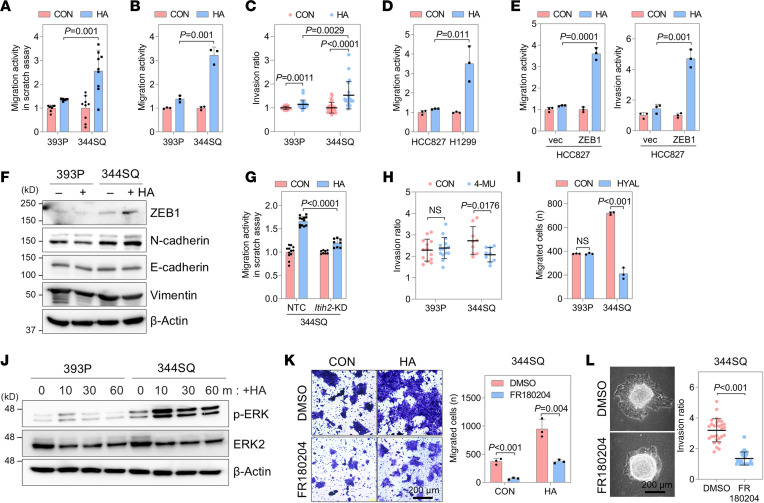
Mesenchymal-like cells are more sensitive to the HA matrix than epithelial-like cells. (**A**) Scratch migration assay in 393P and 344SQ cells treated with HA (2 mg/mL). Mean ± SD (*n* = 12). (**B**) Boyden chamber migration assay in 393P and 344SQ cells treated with HA. (**C**) Spheroid invasion assay of 393P and 344SQ cells treated with HA. The spheroid invasion ratios were calculated by ImageJ after 24 hours of incubation. Mean ± SD (393P control, *n* = 21; 393P with HA, *n* = 25; 344SQ control, *n* = 30; 344SQ with HA, *n* = 21). (**D**) Boyden chamber migration assay in HCC827 and H1299 cells treated with HA. (**E**) Boyden chamber migration (left) and invasion (right) assays in HCC827-vec and HCC827-ZEB1 cells treated with HA. (**F**) Western blot of ZEB1, N-cadherin, E-cadherin, and vimentin in 393P and 344SQ cells treated with HA. (**G**) Scratch migration assay of 344SQ-ITIH2-KD and NTC cells with mean ± SD (*n* = 12). (**H**) Spheroid invasion assay of 393P and 344SQ cells treated with 4-MU (1 mg/mL). Mean ± SD (393P control, *n* = 13; 393P with 4-MU, *n* = 15; 344SQ control, *n* = 9; 344SQ with 4-MU, *n* = 9). (**I**) Boyden chamber migration assay in 393P and 344SQ cells with hyaluronidase (HYAL). (**J**) Western blot of p-ERK and ERK2 in 393P and 344SQ cells with HA treatment. (**K**) Boyden chamber migration assay of 344SQ cells treated with HA and FR180204 (10 μM). Scale bar: 200 μm. (**L**) Spheroid invasion assay of 344SQ cells treated with FR180204. Mean ± SD (DMSO, *n* = 44; FR180204, *n* = 44). Scale bar: 200 μm. Data represent the mean ± SD from a single experiment with biological replicates (*n* = 3, unless otherwise specified) and are representative of at least 3 independent experiments. *P* values determined by 2-tailed Student’s *t* test.

In the TME, cancer cells are ensconced within an HA matrix that aids their migration and invasion. Treatment of lung cancer cells with HA heightened their mobility and invasiveness by inducing partial EMT, particularly evident in mesenchymal-like (high-ZEB1) 344SQ and H1299 cells compared with epithelial-like (low-ZEB1) 393P and HCC827 cells (Figure 5, A–F). Depletion of ITIH2 impeded the migration of 344SQ cells, which was otherwise enhanced by HA treatment (Figure 5G). Inhibition of HA synthesis by 4-MU or hyaluronidase treatment reduced the invasiveness and motility of 344SQ cells, whereas its impact on 393P cells was minimal (Figure 5, H and I). These observations suggest that 344SQ cells, actively forming the HA matrix, depend more on HA for migration and invasion than do 393P cells. The divergent responses may be attributed, at least in part, to the distinct activation of the ERK pathway downstream of HA signaling (34). In 344SQ cells, ERK phosphorylation increased in a time-dependent manner after HA treatment, a response less pronounced in 393P cells (Figure 5J). Additionally, treatment with an ERK inhibitor (FR180204) effectively curbed the migration and invasion of 344SQ cells (Figure 5, K and L). These results indicate that ZEB1 governs the response to HA, with high-ZEB1 cells showing greater sensitivity to extracellular HA compared with low-ZEB1 cells.

### ZEB1 is a transcription activator of HAS2, an HA synthase.

**Figure 6:**
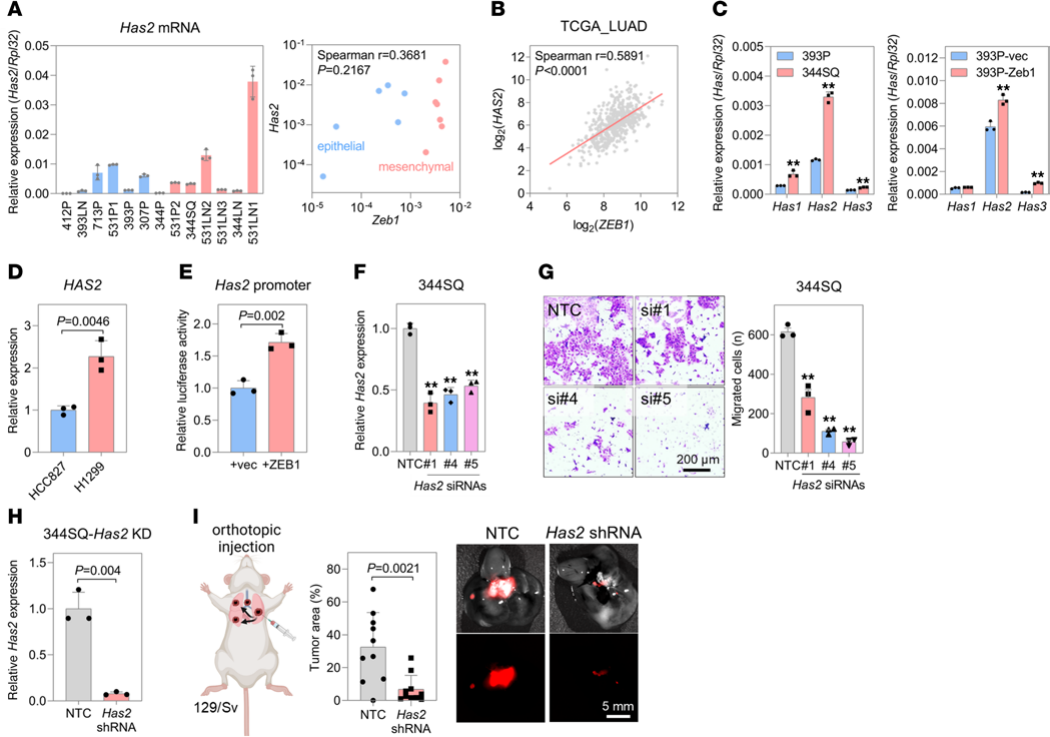
ZEB1 upregulates HAS2 expression. (**A**) qRT-PCR of *Has2* mRNA levels in epithelial-like (blue) and mesenchymal-like (red) murine lung cancer cells. A scatterplot of *Zeb1* and *Has2* mRNA levels is presented (right). (**B**) Scatterplot of *ZEB1* and *HAS2* mRNA levels in TCGA-LUAD data (*n* = 517). (**C**) qRT-PCR of mRNA levels of HAS family members (*Has1*–*Has3*) in 393P, 344SQ, 393P-vec, and 393P-ZEB1 cells. **P < 0.01 by 2-tailed Student’s *t* test. (**D**) qRT-PCR of *HAS2* mRNA levels in HCC827 and H1299 cells. *P* value determined by 2-tailed Student’s *t* test. (**E**) Luciferase reporter assay of *Has2* promoter activity. Murine *Has2* promoter region (2,077 bp; –1,182 to 895 from the transcription start site) was inserted into a luciferase reporter vector (pGL3-Basic). Luciferase reporter was cotransfected with the ZEB1 expression vector. *P* value determined by 2-tailed Student’s *t* test. (**F**) qRT-PCR of *Has2* mRNA levels in 344SQ cells transfected with *Has2* siRNAs (siRNA nos. 1, 4, and 5). **P < 0.01 by 1-way ANOVA followed by Dunnett’s multiple-comparison test. (**G**) Boyden chamber migration assay of 344SQ cells transfected with *Has2* siRNAs. Cells were seeded in upper inserts, and after 24 hours, migrated cells were stained with crystal violet. **P < 0.01 by 1-way ANOVA followed by Dunnett’s multiple-comparison test. Scale bar: 200 μm. (**H**) qRT-PCR of *Has2* mRNA levels in 344SQ cells transduced with *Has2* shRNA. *P* value from 2-tailed Student’s *t* test. (**I**) Mouse orthotopic injection of 344SQ-HAS2-KD cells. Cells labeled with mCherry were injected into the left lung (*n* = 10). After 1 week, lung tumors were measured at necropsy under a fluorescence microscope. *P* value determined by 2-tailed Student’s *t* test. Scale bar: 5 mm. Data represent the mean ± SD from a single experiment with biological replicates (*n* = 3, unless otherwise specified) and are representative of at least 3 independent experiments.

ZEB1 has emerged as a pivotal transcription factor, steering the synthesis and receptor-mediated signaling of HA (35). Our scrutiny of 13 murine lung cancer cell lines and TCGA-LUAD data disclosed a positive correlation between *ZEB1* and *HAS2* mRNA levels, solidifying ZEB1’s role as a transcription activator for HAS2, an HA synthase (Figure 6, A and B). Within the HA synthase family, *Has2* exhibited the highest expression and proved to be under the regulatory influence of ZEB1 in murine lung cancer cells (Figure 6, C and D). In line with previous findings (35), ZEB1 heightened the promoter activity of the *Has2* gene (Figure 6E). ChIP-sequencing data from MCF7 cells corroborated ZEB1’s binding to the promoter region of *HAS2* (Supplemental Figure 1). Depletion of HAS2 in 344SQ cells reduced their motility and invasiveness (Figure 6, F–I), affirming that ZEB1, by upregulating HAS2, promotes HA synthesis and consequently enhances cell migration and invasion.

### ZEB1 controls the splice isoform switching of CD44, an HA receptor.

**Figure 7:**
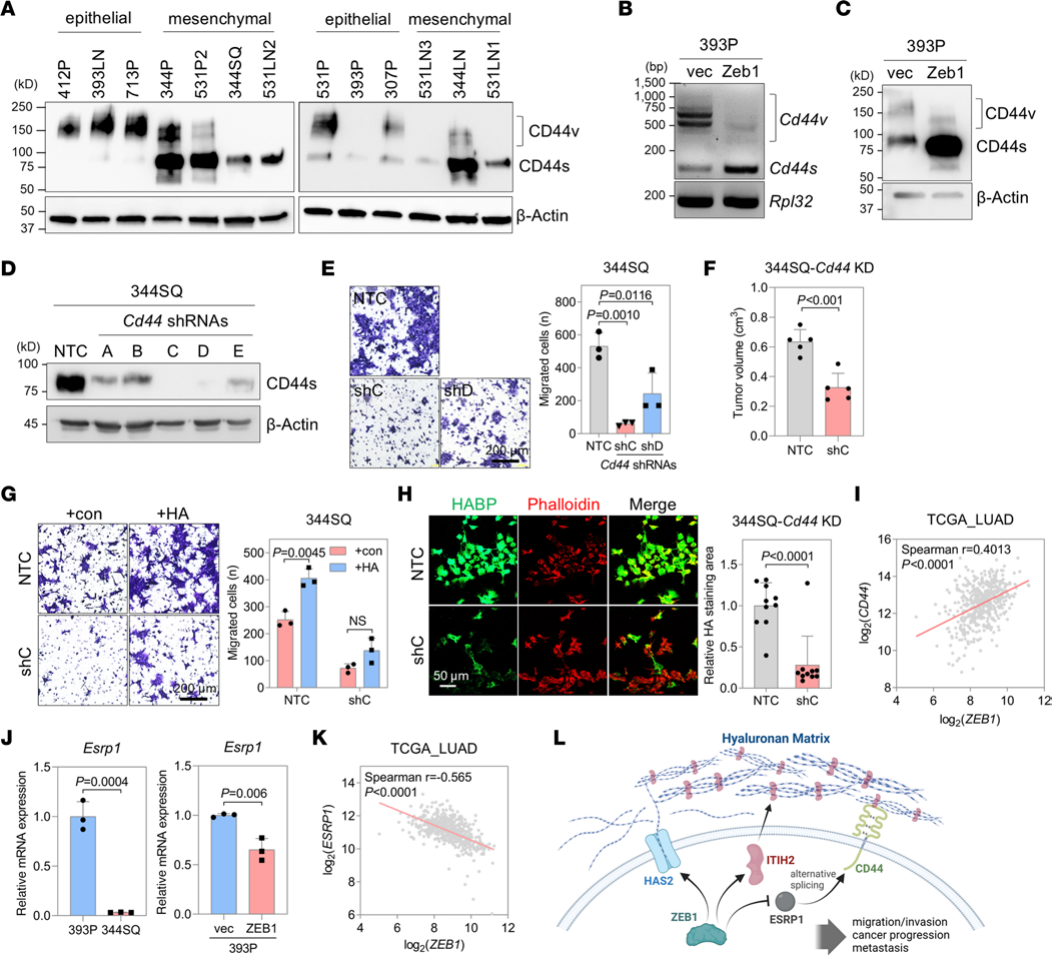
ZEB1 controls CD44 expression. (**A**) Western blot of CD44 in epithelial-like and mesenchymal-like murine lung cancer cells. Standard (s) and variant (v) isoforms of CD44 are indicated. (**B**) RT-PCR of *Cd44* in 393P-vec and 393P-ZEB1 cells. Standard (s) and variant (v) splicing isoforms are indicated. *Rpl32* was used as a loading control. (**C**) Western blot of CD44 in 393P-vec and 393P-ZEB1 cells. (**D**) Western blot of CD44 in 344SQ cells transduced with *Cd44* shRNAs (shA–E). (**E**) Boyden chamber migration assay of 344SQ-CD44-KD (shC and shD) and NTC cells. Cells were seeded in upper inserts, and after 24 hours, migrated cells were stained with crystal violet. *P* values determined by 1-way ANOVA followed by Dunnett’s multiple-comparison test. Scale bar: 200 μm. (**F**) Mouse subcutaneous injection of 344SQ-CD44-KD (shC) and NTC cells. Tumor volume was measured at necropsy. *P* value from 2-tailed Student’s *t* test. (**G**) Boyden chamber migration assay of 344SQ-CD44-KD and NTC cells treated with or without HA. Cells were seeded in upper inserts coated with HA, and after 24 hours, migrated cells were stained with crystal violet. *P* value determined by 2-tailed Student’s *t* test. Scale bar: 200 μm. (**H**) Confocal microscopy of 344SQ-CD44-KD and NTC cells stained with HABP and streptavidin–Alexa Fluor 488 (green) and phalloidin (red). Mean ± SD (NTC, *n* = 10; shC, *n* = 10). Scale bar: 50 μm. (**I**) Scatterplot of *ZEB1* and *CD44* mRNA levels in TCGA-LUAD data (*n* = 517). (**J**) qRT-PCR of *Esrp1* mRNA levels in 393P, 344SQ, 393P-vec, and 393P-ZEB1 cells. (**H**) and (**I**) *P* values determined by 2-tailed Student’s *t* test. (**K**) Scatterplot of *ZEB1* and *ESRP1* mRNA levels in TCGA-LUAD data (*n* = 517). (**L**) Diagram showing the HA network reconstructed by ZEB1. Data represent the mean ± SD from a single experiment with biological replicates (*n* = 3, unless otherwise specified) and are representative of at least 3 independent experiments.

CD44 serves as an HA receptor, engaging in interactions with HA. During the EMT, two splice isoforms of CD44, CD44 standard (CD44s) and CD44 variants (CD44v), exhibit distinct functional characteristics (10, 20, 36). The switch from CD44v to CD44s promotes EMT and contributes to cancer progression (20, 37). In murine lung cancer cells, CD44s expression is higher in mesenchymal-like cells than in epithelial-like cells (Figure 7A). When lung cancer cells were treated with TGF-β, a potent EMT inducer, CD44s expression increased (Supplemental Figure 6A). This increase was restored after TGF-β removal, suggesting that CD44 isoform switching depends on the EMT status.

ZEB1 facilitates the isoform switching from CD44v to CD44s, as demonstrated at both mRNA and protein levels (Figure 7, B and C). This implies that the abundance of CD44s may underlie traits such as heightened migratory and invasive capacities. CD44 KD in 344SQ cells resulted in diminished motility, tumorigenicity, and responsiveness to HA (Figure 7, D–G), indicating CD44’s role in mediating cellular responses to HA. Moreover, CD44 KD led to a reduction in HA matrix formation surrounding lung cancer cells, further affirming the functional importance of CD44 in the HA network (Figure 7H).

In TCGA-LUAD, *CD44* exhibits a positive correlation with *ZEB1* at the mRNA level (Figure 7I). This correlation is attributed to ZEB1’s suppressive effect on ESRP1, a pivotal regulator of CD44 alternative splicing (20, 37). The mRNA levels of *Esrp1* were lower in 344SQ cells and 393P-ZEB1 cells than in 393P and 393P-vector cells (Figure 7J). In TCGA-LUAD data, *ESRP1* shows a negative correlation with *ZEB1* at the mRNA level (Figure 7K). ChIP-sequencing data in MCF7 cells confirm ZEB1’s binding to the promoter region of *ESRP1* (Supplemental Figure 1). These findings collectively suggest that ZEB1 orchestrates the HA network at the receptor level by regulating the splice isoform switching of CD44. ZEB1 was found to modulate the expression of other HA-binding proteins and receptors, which warrants further study (Supplemental Figure 6B). In contrast, other EMT-inducing transcription factors, such as ZEB2, SNAIL, SLUG (Snail2), and TWIST, exhibited little or no effect on *Itih2* and *Has2* expression or on the formation of the extracellular HA matrix (Supplemental Figure 6, C–I). Overall, ZEB1 plays a pivotal role in governing the comprehensive formation of the HA network, encompassing HA synthesis, matrix formation, and receptor-mediated signaling (Figure 7L).

### Sincalide suppresses lung cancer progression by inhibiting ITIH2.

**Figure 8:**
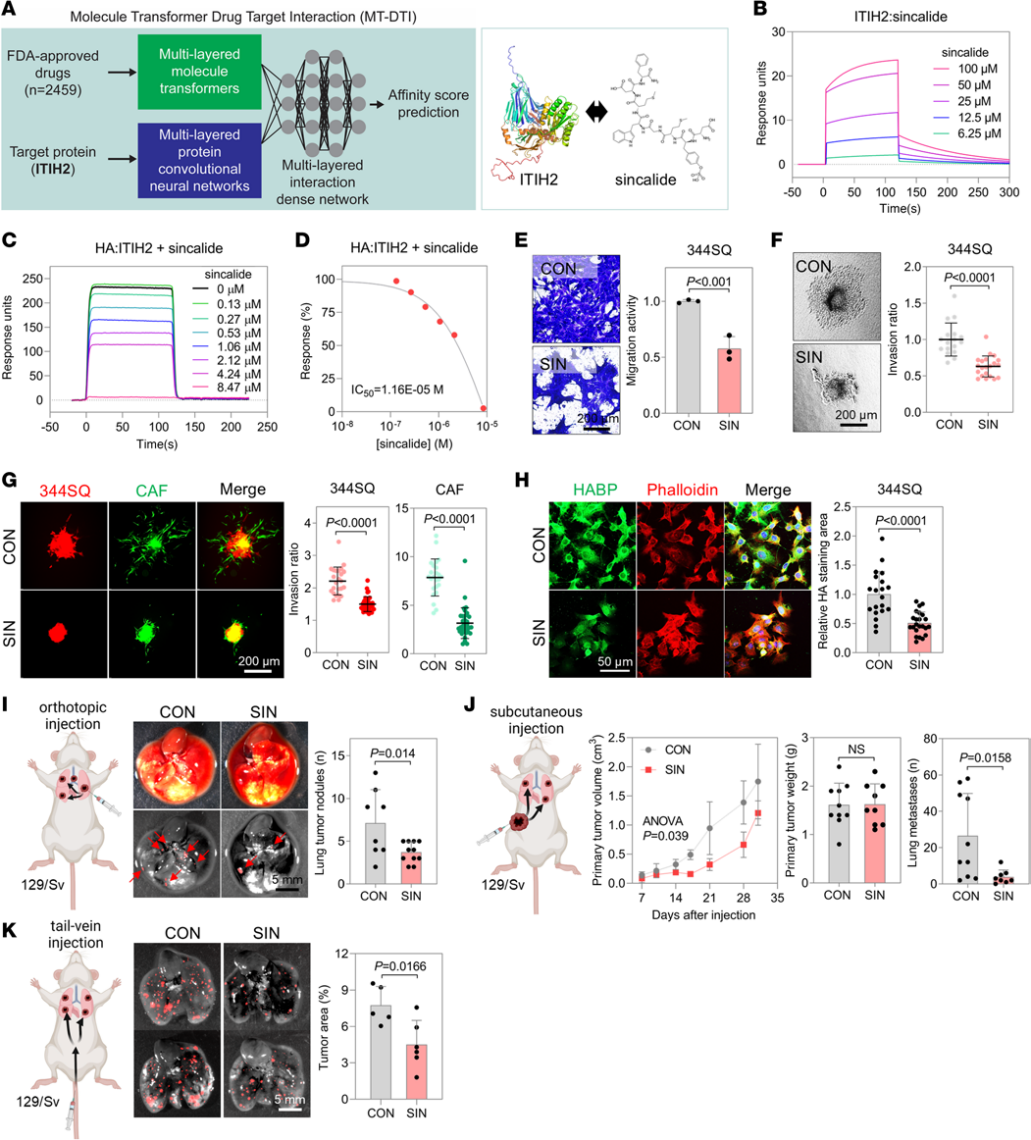
Sincalide, an ITIH2 inhibitor, inhibits the migration and invasion of lung cancer cells. (**A**) Prediction of ITIH2 inhibitors using the MT-DTI algorithm. (**B**) Surface plasmon resonance (SPR) analysis showing binding of sincalide to the ITIH2 protein. (**C** and **D**) SPR analysis showing inhibition of binding between HA and the ITIH2 protein by sincalide. (**E** and **F**) Boyden chamber migration (**E**) and spheroid invasion assay (**F**) of 344SQ cells treated with sincalide (SIN). Scale bar: 200 μm. (**G**) Spheroid invasion assay of CAFs and 344SQ cells treated with sincalide. Mean ± SD (CON, *n* = 23; SIN, *n* = 32). Scale bar: 200 μm. (**H**) Confocal microscopy of 344SQ cells treated with sincalide. Mean ± SD (CON, *n* = 20; SIN, *n* = 24). Scale bar: 50 μm. (**I**) 344SQ cells were orthotopically injected into the left lung (*n* = 8 or 9). Sincalide (2.5 mg/kg of body weight) was injected intraperitoneally twice a week until mice were euthanized. After 7 days, the number of lung tumor nodules was measured at necropsy. Scale bar: 5 mm. (**J**) 344SQ cells were subcutaneously injected (*n* = 9), and primary tumor volumes (left graph) were measured until mice were euthanized. After 6 weeks, tumor weights (middle graph) and number of lung metastases (right graph) were measured at necropsy. (**K**) 344SQ cells were injected via tail vein (*n* = 5 or 6). After 10 days, tumor nodules colonized in lungs were assessed. Scale bar: 5 mm. Data represent the mean ± SD from a single experiment with biological replicates (*n* = 3, unless otherwise specified) and are representative of at least 3 independent experiments. P values determined by 2-tailed Student’s t test (**E**–**I**, **J** [right 2 bar graphs], and **K**) or 2-way ANOVA test (**J** [left]).

Given the pivotal role of ITIH2 in cancer cell invasion and metastasis, directing therapeutic interventions toward ITIH2 poses a promising strategy. Employing the Molecule Transformer Drug-Target Interaction (MT-DTI) algorithm, a deep learning model for drug-target interactions (Figure 8A) (38), we screened 2,459 FDA-approved drugs to identify candidates disrupting the ITIH2-HA interaction and impeding HA matrix formation (Supplemental Figure 7A). Sincalide, also known as CCK-8, stood out as the most effective compound, displaying inhibitory effects on lung cancer cell migration and invasion without causing severe cytotoxicity upon experimental validation (Supplemental Figure 7, B–F).

Sincalide, primarily used for diagnosing gallbladder and pancreatic disorders, stimulates gallbladder contractions and induces digestive enzyme release from the pancreas (39). Surface plasmon resonance analysis confirmed its effective binding to ITIH2 (Figure 8B), preventing its interaction with HA (Figure 8, C and D). Sincalide exhibited inhibitory effects on the migration and invasion of various cancer cells (lung, head and neck, breast, and prostate cancer) (Figure 8, E and F, and Supplemental Figure 7, G and H) and reduced CAF invasion in a coculture assay (Figure 8G). Furthermore, sincalide mitigated the formation of the HA matrix surrounding 344SQ cells (Figure 8H). Unlike anti-survival/anti-proliferation drugs such as cisplatin and EGFR inhibitors (40, 41), ZEB1 did not drive resistance to sincalide (Supplemental Figure 7, I and J). Instead, ZEB1 increased sensitivity to sincalide (Supplemental Figure 7K).

Hypothesizing that disrupting the HA matrix crucial for cancer cell migration and invasion would diminish lung cancer progression, we administered sincalide intraperitoneally or intravenously in syngeneic 129/Sv mice. In the orthotopic injection model, sincalide markedly suppressed local invasion of lung cancer cells (Figure 8I and Supplemental Figure 8A). While primary tumor growth in the subcutaneous injection model remained unaffected (Supplemental Figure 8, B–E), sincalide effectively curtailed lung metastasis (Figure 8J). In a tail-vein injection model, sincalide attenuated the lung colonization of 344SQ cells (Figure 8K). During the treatment period, sincalide had a minimal impact on body weight in comparison with the control group (Supplemental Figure 9A). Hematological analysis, including complete blood count parameters, as well as ELISA tests for plasma aspartate aminotransferase, alanine transaminase, and blood urea nitrogen levels, along with H&E staining of the major organs, confirmed that sincalide had no adverse effects on the mice at the concentration used in this study (2.5 mg/kg of body weight; Supplemental Figure 9, B–D). Even when the dosage was doubled to 5 mg/kg, the antitumor effect of sincalide remained comparable to that of the original dosage, suggesting that the original dosage is sufficient to fully elicit the antitumor activity of sincalide (Supplemental Figure 10, A and B). The combination of sincalide and 4-MU showed no synergistic effects, probably because they both act on the same therapeutic target (Supplemental Figure 10C). Since sincalide disrupts HA matrix formation downstream of HA synthesis, further blocking HA synthesis with 4-MU may not enhance inhibition. Sincalide treatment slightly inhibited spontaneous lung tumor growth in *Kras^G12D^* mice, though this effect was statistically insignificant, consistent with the results observed in the syngeneic mouse models (Supplemental Figure 10D). We were unable to assess sincalide’s impact on metastasis, as *Kras^G12D^* mice rarely develop metastases (42). Furthermore, sincalide treatment suppressed CAF activation and M2 macrophage recruitment (Supplemental Figure 10E), thereby hindering cancer progression in the TME. These findings underscore sincalide’s role in inhibiting cancer progression by disrupting the ITIH2-HA interaction, leading to a reduction in HA matrix formation crucial for lung cancer cell migration and invasion.

## Discussion

In this investigation, we have brought to light the pivotal role of ZEB1 in the regulation and remodeling of the HA network. ZEB1 instigates the transcriptional activation of crucial components within this network, specifically ITIH2, HAS2, and CD44. Consequently, this process promotes cross-linking, synthesis, and intracellular signaling in the HA matrix. The remodeled HA matrix, orchestrated by ZEB1, facilitates the migration and invasion of cancer cells. Furthermore, the effective suppression of lung cancer progression was demonstrated by blocking of HA matrix formation using an ITIH2 inhibitor.

ZEB1’s multifaceted nature is evident in its induction of EMT (20), remodeling of the cytoskeleton and ECM (24), pro-metastatic Golgi stacking (43), facilitation of secretory and endocytic vesicular trafficking (44), and regulation of drug resistance and immune suppression (45, 46). This versatility arises from ZEB1’s capability to promote or suppress the transcription of various genes, including microRNAs and long noncoding RNAs (23, 24, 47), through interactions with corepressors and coactivators like CtBP and p300 (48). In this study, we propose another function for ZEB1 as a comprehensive regulator of the HA network. Beyond confirming the previously reported ZEB1-induced transcriptional regulation of the HA synthase HAS2 (35) and HA receptor CD44 (20), we provide evidence that ZEB1 stimulates the transcription of the HA-binding protein ITIH2. ZEB1-driven remodeling of the HA network plays a pivotal role in cancer cell migration and invasion. Moreover, our findings indicate that sincalide, a specific ITIH2 inhibitor identified through deep learning–based screening, has the potential to disrupt the HA matrix, thereby preventing cancer progression.

The findings presented here suggest that the response to HA is more prominent in mesenchymal-like cancer cells compared with epithelial-like cancer cells. HA receptors, such as CD44, along with its downstream signaling pathways like ERK/MAPK, play a crucial role in regulating the cancer cells’ response to HA. Notably, the expression of the standard isoform of CD44 was upregulated by ZEB1, and ERK activation was elevated in mesenchymal-like cancer cells. However, HA-mediated cell migration and invasion may also be regulated by other HA receptors and signaling pathways. Upon examining the expression levels of 2 additional HA receptors, RHAMM (encoded by the *Hmmr* gene) and LYVE-1 (encoded by the *Lyve1* gene), we found that LYVE-1 expression was also elevated in mesenchymal cancer cells, whereas RHAMM expression was slightly higher in epithelial cancer cells (Supplemental Figure 6J). In HT1080 fibrosarcoma cells, RHAMM regulates cell proliferation through a β-catenin/c-myc signaling axis (49), and CD44 can influence gastric cancer cell growth by interacting with FGFR2 and c-myc (50). The RHAMM receptor and c-myc signaling can be active in epithelial cancer cells and may regulate other HA-mediated cellular responses.

ZEB1 is known to regulate ECM stability and composition by controlling fibulin-2 (51), lysyl oxidases (52), and lysyl hydroxylase 2 (53), thereby facilitating cancer cell migration and invasion. Although ITIH2 KD had minimal effect on the expression of these ECM-related factors (Supplemental Figure 6K), ITIH2 (or the HA network) may modulate other ECM components to reconstruct a pro-invasive TME. In addition, further analysis of TCGA-LUAD and ENCODE data revealed that HNF4A may also be a candidate transcription factor that promotes ITIH2 expression (Supplemental Figure 11), which warrants further investigation.

As demonstrated in this study, the impact of the HA matrix on cancer cell migration and invasion underscores its efficacy as a target for cancer inhibition. Hyaluronidases have shown promise in inhibiting tumor growth (54), enhancing effectiveness when combined with other anticancer strategies (55), and promoting intratumoral T cell infiltration (56). The HAS inhibitor 4-MU exhibits anticancer activity against various cancer types (57). However, direct degradation or inhibition of HA production may disrupt HA equilibrium in the microenvironment, potentially leading to unforeseen outcomes within tumors and normal tissue contexts. Here, we introduce another method for inhibiting HA function using the ITIH2 inhibitor sincalide. This inhibitor hinders the formation of the HA matrix or cables outside cancer cells without causing destructive degradation or depletion of HA. By focusing on ITIH2, this approach allows finer control over the cellular microenvironment, offering a potentially more efficient therapeutic strategy for cancer.

ITIH2 was initially identified as a serum-based protein covalently linked to HA (58). The ITIH family comprises 5 members (ITIH1–5), all associated with cancer progression. *ITIH2* mRNA is downregulated in multiple solid tumors (59), while *ITIH1* mRNA undergoes downregulation in hepatocellular carcinoma (60). Conversely, ITIH5, induced by p53, suppresses melanoma cell growth and metastasis (61). In contrast, the ITIH-HA complex positively correlates with lymphovascular space invasion, serving as a valuable prognostic marker for predicting disease recurrence in patients with ovarian or endometrial cancer (62, 63). In an idiopathic pulmonary fibrosis model, ITIH2 facilitates neovascularization and lung fibrosis (64), suggesting an analogous role in angiogenesis within the lung TME. In this study, we demonstrated a correlation between ITIH2 expression and ZEB1 expression, as well as EMT status, in LUAD. Furthermore, our findings revealed that ITIH2, in conjunction with the HA matrix, facilitates the motility and invasiveness of lung cancer cells, supporting their pro-invasive role within the lung TME. Further studies are needed to understand better the specific roles of ITIH2 and the HA matrix at each metastasis stage, including primary tumor dissemination, lymph node invasion, circulation, and colonization.

Focusing on the pro-invasive function of ITIH2, we employed a deep learning algorithm to repurpose FDA-approved drugs as potential ITIH2 inhibitors, identifying promising candidates for inhibiting ITIH2 and acting as anticancer agents. Sincalide, an 8–amino acid C-terminal fragment of cholecystokinin, is extensively used in cholescintigraphy for inducing gallbladder contractions and stimulating bile secretion without severe side effects (39). While prior evidence indicates that sincalide can relieve ileus symptoms in patients with cancer undergoing vincristine treatment (65), its anticancer effects or application in the management of patients with cancer lacked support. In this study, sincalide demonstrated minimal toxicity in cell and mouse models, effectively restraining cancer cell migration, invasion, and cancer progression. These preclinical findings strongly indicate its potential as an anticancer treatment. Further research is imperative to determine its optimal usage, dosage, and mechanisms of action.

ITIH2 plays an important role in the formation of the extracellular HA matrix and cables, actively promoting cellular motility and invasiveness. These HA matrices and cables play a role in EV secretion and transport (32). Moreover, HA-coated EVs derived from cancer cells demonstrate improved delivery to neighboring target cells (31). Our study suggests that EV binding and uptake are particularly active in cells with a well-established HA matrix, and HA cables may contribute to EV transport. Given the growing clinical importance of EVs in cell-cell interactions within the TME, it is crucial to consider the impact of the HA network regulated by ZEB1 and ITIH2 on the production and transport of EVs.

In conclusion, ZEB1 orchestrates cancer cell migration and invasion through comprehensive regulation of the HA network, including ITIH2. Strategies to inhibit the pro-invasive HA network open up possibilities for advancing cancer treatment strategies.

## Methods

See Supplemental Methods for full details.

### Sex as a biological variable.

Our study included both male and female mice, and we observed similar findings across both sexes.

### Cell culture studies.

Murine lung cancer cell lines generated from lung tumors in *Kras^LA1/+^ Trp53^R172H/+^* mice and human lung cancer cell lines were cultured in RPMI 1640 medium (Welgene) supplemented with 10% fetal bovine serum (FBS; HyClone). Lung CAFs were maintained in α-MEM (Welgene) containing 10% FBS, penicillin/streptomycin (100 U/mL and 100 μg/mL; Welgene), 2 mM l-glutamine (Welgene), and 1 mM sodium pyruvate (Welgene). Using these cells, migration and invasion assays were performed.

### Fluorescent cellular imaging.

HA was stained using biotinylated HA-binding protein (HABP; HKD-BC41, AMSBIO) according to the manufacturer’s protocol.

### Mouse experiments.

Mouse experiments with syngeneic (129/Sv) mice were performed as described in Supplemental Methods, following the Institutional Animal Care and Use Committee (IACUC) guidelines.

### Statistics.

Data values are represented as mean ± SD unless stated otherwise. GraphPad Prism was used for statistical analyses. Two-group comparisons were performed using a 2-tailed *t* test when the data were normally distributed. Multiple comparisons were performed using 1-way ANOVA followed by Dunnett’s multiple-comparison test. Differences were considered statistically significant when *P* values were less than 0.05. Experiments were performed 3–4 times unless otherwise stated. Statistical significance is shown as follows: *P < 0.05; **P < 0.01.

### Study approval.

The mouse studies were approved by the IACUC of Ewha Womans University College of Medicine (EWHA MEDIACUC 23-030-t) before the start of the research. All procedures involving the mice, including treatment and euthanasia, were conducted according to IACUC guidelines.

### Data availability.

All data associated with this study are present in the paper or the supplemental materials.

## Author contributions

SL, JMK, and YHA designed the conceptual framework of this study. SL, JP, SC, EJK, SO, YL, DHS, SYK, and YHA performed experiments in vitro and in vivo. SP and KK designed an algorithm to predict ITIH2 inhibitors. SL and YHA drafted and revised the manuscript. All the authors read and approved the final version of the manuscript.

## Supplementary Material

Supplemental data

Unedited blot and gel images

Supporting data values

## Version 1 04/03/2025

In-Press Preview

## Version 2 06/02/2025

Electronic publication

## References

[1] Winkler J (2020). Concepts of extracellular matrix remodelling in tumour progression and metastasis. Nat Commun.

[2] Karousou E (2023). Hyaluronan in the cancer cells microenvironment. Cancers (Basel).

[3] Gatti V (2018). ΔNp63 regulates the expression of hyaluronic acid-related genes in breast cancer cells. Oncogenesis.

[4] Kim PK (2021). Hyaluronic acid fuels pancreatic cancer cell growth. Elife.

[5] Brichkina A (2016). p38MAPK builds a hyaluronan cancer niche to drive lung tumorigenesis. Genes Dev.

[6] Tahkola K (2021). Stromal hyaluronan accumulation is associated with low immune response and poor prognosis in pancreatic cancer. Sci Rep.

[7] Toole BP, Slomiany MG (2008). Hyaluronan: a constitutive regulator of chemoresistance and malignancy in cancer cells. Semin Cancer Biol.

[8] McAtee CO (2014). Emerging roles for hyaluronidase in cancer metastasis and therapy. Adv Cancer Res.

[9] Hamilton SR (2007). The hyaluronan receptors CD44 and Rhamm (CD168) form complexes with ERK1,2 that sustain high basal motility in breast cancer cells. J Biol Chem.

[10] Witschen PM (2020). Tumor cell associated hyaluronan-CD44 signaling promotes pro-tumor inflammation in breast cancer. Cancers (Basel).

[11] Song JM (2019). Hyaluronan-CD44/RHAMM interaction-dependent cell proliferation and survival in lung cancer cells. Mol Carcinog.

[12] Bourguignon LY (2002). Hyaluronan promotes signaling interaction between CD44 and the transforming growth factor beta receptor I in metastatic breast tumor cells. J Biol Chem.

[13] Sun Y (2022). FGF9 promotes expression of HAS2 in palatal elevation via the Wnt/β-Catenin/TCF7L2 pathway. Biomolecules.

[14] Monzón ME (2008). Hyaluronidase expression and activity is regulated by pro-inflammatory cytokines in human airway epithelial cells. Am J Respir Cell Mol Biol.

[15] Zhang G (2021). Reduced hyaluronan cross-linking induces breast cancer malignancy in a CAF-dependent manner. Cell Death Dis.

[16] Kobayashi N (2010). Hyaluronan deficiency in tumor stroma impairs macrophage trafficking and tumor neovascularization. Cancer Res.

[17] Kalluri R, Weinberg RA (2009). The basics of epithelial-mesenchymal transition. J Clin Invest.

[18] Koistinen V (2017). EMT induced by EGF and wounding activates hyaluronan synthesis machinery and EV shedding in rat primary mesothelial cells. Matrix Biol.

[19] Porsch H (2013). Efficient TGFβ-induced epithelial-mesenchymal transition depends on hyaluronan synthase HAS2. Oncogene.

[20] Larsen JE (2016). ZEB1 drives epithelial-to-mesenchymal transition in lung cancer. J Clin Invest.

[21] Voutouri C, Stylianopoulos T (2018). Accumulation of mechanical forces in tumors is related to hyaluronan content and tissue stiffness. PLoS One.

[22] Bota-Rabassedas N (2021). Contextual cues from cancer cells govern cancer-associated fibroblast heterogeneity. Cell Rep.

[23] Gibbons DL (2009). Contextual extracellular cues promote tumor cell EMT and metastasis by regulating miR-200 family expression. Genes Dev.

[24] Ahn YH (2012). ZEB1 drives prometastatic actin cytoskeletal remodeling by downregulating miR-34a expression. J Clin Invest.

[25] Padhye A (2019). A novel ex vivo tumor system identifies Src-mediated invasion and metastasis in mesenchymal tumor cells in non-small cell lung cancer. Sci Rep.

[26] Ungewiss C (2016). The microRNA-200/Zeb1 axis regulates ECM-dependent β1-integrin/FAK signaling, cancer cell invasion and metastasis through CRKL. Sci Rep.

[27] Győrffy B (2013). Online survival analysis software to assess the prognostic value of biomarkers using transcriptomic data in non-small-cell lung cancer. PLoS One.

[28] Zhao X (2024). FBXO11 mediates ubiquitination of ZEB1 and modulates epithelial-to-mesenchymal transition in lung cancer cells. Cancers (Basel).

[29] Kultti A (2009). 4-Methylumbelliferone inhibits hyaluronan synthesis by depletion of cellular UDP-glucuronic acid and downregulation of hyaluronan synthase 2 and 3. Exp Cell Res.

[30] Rho JK (2014). MET and AXL inhibitor NPS-1034 exerts efficacy against lung cancer cells resistant to EGFR kinase inhibitors because of MET or AXL activation. Cancer Res.

[31] Babula A (2023). CD44‑hyaluronan axis plays a role in the interactions between colon cancer‑derived extracellular vesicles and human monocytes. Oncol Lett.

[32] Rilla K (2014). Hyaluronan-coated extracellular vesicles—a novel link between hyaluronan and cancer. Adv Cancer Res.

[33] Aaltonen N (2022). MCF10CA breast cancer cells utilize hyaluronan-coated EV-rich trails for coordinated migration. Front Oncol.

[34] Serbulea M (1999). Hyaluronan activates mitogen-activated protein kinase via Ras-signaling pathway. Int J Oncol.

[35] Preca BT (2017). A novel ZEB1/HAS2 positive feedback loop promotes EMT in breast cancer. Oncotarget.

[36] Bourguignon LY (2014). Hyaluronan-CD44 interaction promotes oncogenic signaling, microRNA functions, chemoresistance, and radiation resistance in cancer stem cells leading to tumor progression. Adv Cancer Res.

[37] Brown RL (2011). CD44 splice isoform switching in human and mouse epithelium is essential for epithelial-mesenchymal transition and breast cancer progression. J Clin Invest.

[38] Shin B (2019). Dynamically personalized detection of hemorrhage. Proc Mach Learn Res.

[39] Ziessman HA (2019). Sincalide: a review of clinical utility, proper infusion methodology, and alternative cholecystogogues. J Nucl Med Technol.

[40] Zhang T (2016). A genetic cell context-dependent role for ZEB1 in lung cancer. Nat Commun.

[41] Wu Y (2019). UBE2C induces cisplatin resistance via ZEB1/2-dependent upregulation of ABCG2 and ERCC1 in NSCLC Cells. J Oncol.

[42] Johnson L (2001). Somatic activation of the K-ras oncogene causes early onset lung cancer in mice. Nature.

[43] Tan X (2017). Epithelial-to-mesenchymal transition drives a pro-metastatic Golgi compaction process through scaffolding protein PAQR11. J Clin Invest.

[44] Banerjee P (2021). The EMT activator ZEB1 accelerates endosomal trafficking to establish a polarity axis in lung adenocarcinoma cells. Nat Commun.

[45] Chen L (2014). Metastasis is regulated via microRNA-200/ZEB1 axis control of tumour cell PD-L1 expression and intratumoral immunosuppression. Nat Commun.

[46] Meidhof S (2015). ZEB1-associated drug resistance in cancer cells is reversed by the class I HDAC inhibitor mocetinostat. EMBO Mol Med.

[47] Kim EJ (2022). ZEB1-regulated lnc-Nr2f1 promotes the migration and invasion of lung adenocarcinoma cells. Cancer Lett.

[48] Caramel J (2018). Pleiotropic roles for ZEB1 in cancer. Cancer Res.

[49] Kouvidi K (2016). Receptor for hyaluronic acid- mediated motility (RHAMM) regulates HT1080 fibrosarcoma cell proliferation via a β-catenin/c-myc signaling axis. Biochim Biophys Acta.

[50] Park J (2016). A reciprocal regulatory circuit between CD44 and FGFR2 via c-myc controls gastric cancer cell growth. Oncotarget.

[51] Baird BN (2013). Fibulin-2 is a driver of malignant progression in lung adenocarcinoma. PLoS One.

[52] Peng DH (2017). ZEB1 induces LOXL2-mediated collagen stabilization and deposition in the extracellular matrix to drive lung cancer invasion and metastasis. Oncogene.

[53] Liu W (2018). Lysyl hydroxylases are transcription targets for GATA3 driving lung cancer cell metastasis. Sci Rep.

[54] Shuster S (2002). Hyaluronidase reduces human breast cancer xenografts in SCID mice. Int J Cancer.

[55] Morosi L (2021). PEGylated recombinant human hyaluronidase (PEGPH20) pre-treatment improves intra-tumour distribution and efficacy of paclitaxel in preclinical models. J Exp Clin Cancer Res.

[56] Farrera-Sal M (2021). Hyaluronidase expression within tumors increases virotherapy efficacy and T cell accumulation. Mol Ther Oncolytics.

[57] Vitale DL (2021). Targeting the tumor extracellular matrix by the natural molecule 4-methylumbelliferone: a complementary and alternative cancer therapeutic strategy. Front Oncol.

[58] Huang L (1993). A serum-derived hyaluronan-associated protein (SHAP) is the heavy chain of the inter alpha-trypsin inhibitor. J Biol Chem.

[59] Hamm A (2008). Frequent expression loss of Inter-alpha-trypsin inhibitor heavy chain (ITIH) genes in multiple human solid tumors: a systematic expression analysis. BMC Cancer.

[60] Chang QH (2021). Pan-cancer analysis identifies *ITIH1* as a novel prognostic indicator for hepatocellular carcinoma. Aging (Albany NY).

[61] Liu J (2021). ITIH5, a p53-responsive gene, inhibits the growth and metastasis of melanoma cells by downregulating the transcriptional activity of KLF4. Cell Death Dis.

[62] Yabushita H (2011). Clinicopathological role of serum-derived hyaluronan-associated protein (SHAP)-hyaluronan complex in endometrial cancer. Obstet Gynecol Int.

[63] Obayashi Y (2008). Role of serum-derived hyaluronan-associated protein-hyaluronan complex in ovarian cancer. Oncol Rep.

[64] Garantziotis S (2008). Serum inter-alpha-trypsin inhibitor and matrix hyaluronan promote angiogenesis in fibrotic lung injury. Am J Respir Crit Care Med.

[65] Jackson DV (1982). Treatment of vincristine-induced ileus with sincalide, a cholecystokinin analog. Cancer Chemother Pharmacol.

[66] Győrffy B (2024). Transcriptome-level discovery of survival-associated biomarkers and therapy targets in non-small-cell lung cancer. Br J Pharmacol.

