# Supplementary material for: Hyaluronan network remodeling by ZEB1 and ITIH2 enhances the motility and invasiveness of cancer cells
Source: J Clin Invest. 2025 Apr 3;135(11):e180570. doi: 10.1172/JCI180570 (PMC12126249; doi:10.1172/JCI180570)
Supplement: Supplemental data [file jci-135-180570-s065.pdf]

Supplemental Materials

Hyaluronan network remodeling by ZEB1 and ITIH2 enhances the motility and invasiveness of cancer cells

Sieun Lee, Jihye Park, Seongran Cho, Eun Ju Kim, Seonyeong Oh, Younseo Lee, Sungsoo Park, Keunsoo Kang, Dong Hoon Shin, Song Yi Ko, Jonathan M. Kurie, Young-Ho Ahn

Supplemental Methods

Cell culture studies

Murine lung cancer cell lines (344SQ, 393P, 307P, 412P, 393LN, 713P, 344P, 344LN, 531P1, 531P2, 531LN1, 531LN2, and 531LN3) were generated from lung tumors in *Kras*$^{LA1/+}$;*Trp53*$^{R172H/+}$ mice as previously described (1). Human lung cancer cell lines (HCC827, H1299, and A549) were purchased from ATCC. Lung cancer cells were cultured in RPMI1640 (Welgene) supplemented with 10% fetal bovine serum (FBS; HyClone) at 37°C in the presence of 5% CO_2. Lung CAFs were isolated as previously described (2) and maintained in alpha-MEM (Welgene) containing 10% FBS, penicillin/streptomycin (100 U/mL and 100 µg/mL, Welgene), 2 mM L-glutamine (Welgene), and 1 mM sodium pyruvate (Welgene). 344SQ cells and CAFs were stably labeled with mCherry and GFP as previously detailed (2). shRNAs targeting *Itih2*, *Zeb1, Has2,* and *Cd44* in pLKO.1 vectors were obtained from Sigma–Aldrich. 344SQ cells were infected with lentiviruses generated from 293T cells that were co-transfected with the lentiviral vectors pMD2.G and psPAX2 (gifts from Didier Trono; Addgene plasmid #12259 and #12260, respectively). After 2–3 days of infection, the cells were selected with 10 µg/mL puromycin (InvivoGen) for over 2 weeks. Human *ITIH2* cDNA was obtained from GeneScript (#OHu30923) and subcloned into pLVX-Blast, modified from the pLVX-Puro vector (Clontech). *Has2*, *Zeb2*,

Snai2, Snai1, and *Twist1* siRNAs were purchased from Bioneer and transfected into 344SQ cells using the TransIT-X2 Dynamic Delivery System (Mirus Bio). Sincalide (Selleckchem) was treated to cells at the concentration of 10 μM for 24 h to block ITIH2 in migration and invasion assays. Cell viability was assessed using the Quanti-Max WST-8 Cell Viability Assay Kit (BIOMAX) following the manufacturer's instructions.

Quantitative reverse transcription PCR

Total RNA was isolated from the cells using the XENOPURE Total RNA Purification Kit (Xenohelix) following the manufacturer's protocol. For mRNA-level analysis, quantitative reverse transcription-PCR (qRT-PCR) assays were conducted using a BioFACT A-Star Real-time PCR Kit with SFCgreen I (BioFACT) after reverse transcription with TOPscript RT DryMIX (Enzynomics). mRNA levels were normalized to *Rpl32* mRNA. For conventional RT-PCR to detect *Cd44* standard and variant mRNAs, reverse-transcribed cDNA templates were amplified using Lamp Taq DNA Polymerase (BioFACT). Specific primers were designed to target exon 6 and exon 18 within murine *Cd44* mRNA. The qRT-PCR primer sequences used in this study are listed in Supplemental Table 1.

Western blotting

Cell lysates were prepared using RIPA lysis buffer (50 mM Tris-Cl pH 7.4, 150 mM NaCl, 1 mM EDTA, and 1% Triton X-100) supplemented with protease inhibitors (Sigma–Aldrich). Subsequently, proteins (50 μg) were separated through SDS-PAGE, transferred onto PVDF membranes (Bio-Rad), and subjected to incubation with primary antibodies and HRP-conjugated secondary antibodies (Bio-Rad). Protein bands were visualized using a Miracle-Star Western Blot Detection System (iNtRON Biotechnology). Antibodies used in this study are listed in Supplemental Table 2.

Migration assays

For the Transwell migration assay, lung cancer cells (1×10^5 cells per well) were seeded in hanging inserts (Labselect) in serum-free medium, and a complete medium with 10% FBS was added to the bottom wells. The cells were then incubated at 37°C for 24 h, and the migrated cells were stained with 0.1% crystal violet. For co-culture assays, a mixture of red-labeled cancer cells (5×10^4 cells per well) and green-labeled CAFs (5×10^4 cells per well) were seeded in hanging inserts, and the migrated cells were observed under a fluorescence microscope. Three randomly selected microscopic fields (×100 magnification) per chamber were captured and counted manually. For the scratch assay, artificial scratches were created using a pipette tip on confluent cell monolayers in six-well plates. The scratch area was subsequently measured at specified time points using ImageJ software (NIH). To examine the effect of HA on cell migration, the cells were treated with HA (2 mg/mL; #HA15M, Lifecore Biomedical) or chondroitin (Sigma–Aldrich) as a control.

Spheroid invasion assays

For spheroid formation, a mixture of lung cancer cells (5×10^4 cells) labeled with mCherry and GFP-labeled fibroblasts (1×10^5 cells) in 5 mL of complete medium containing 20% METHOCEL (Sigma-Aldrich) and 1% Matrigel (BD Biosciences) was suspended on the lids of 150 mm dishes and incubated at 37°C for 2 days. Subsequently, the spheroid mixture (consisting of spheroids in 0.5× PBS, 0.01 N NaOH, and 3 mg/mL collagen) was implanted in the center of each well in a 12-well plate. Once the gels were polymerized, the wells were filled with the cell culture medium. Following 1–2 days, phase-contrast and fluorescence microscopy were used to capture images of the invading cells, and the invasion ratio was calculated by dividing the total area invaded by the central spheroid area using ImageJ software. In addition,

for spheroid overlay cultures, 344SQ-cancer cell spheroids (labeled with red) were placed on top of a confluent layer of CAFs (labeled with green), which were cultured for 24 h prior to the spheroid seeding, and invasive patterns were observed under a confocal microscope (LSM800; ZEISS).

Promoter assays

For promoter luciferase assays, murine *Itih2* (1,889 bp; −1,671–218 from the transcription start site) and *Has2* (2,077 bp; −1,182–895 from the transcription start site) promoter regions were amplified using PCR from 344SQ genomic DNA and inserted into the pGL3-Basic vector (Promega). ZEB1-binding sites in the promoter region were predicted using JASPAR (3) and TFBIND (4). To examine the impact of ZEB1-binding sites, a series of deletions and mutations were introduced. 344SQ cells were then transfected with promoter reporters and pcDNA3.1-ZEB1, with the pCI-neo-hRL vector (Renilla luciferase) serving as an internal control. After 48 h, the luciferase activity was measured using a Duo-Luciferase Assay Kit (GeneCopoeia). The ChIP assay of ZEB1 was performed as described previously (5).

Enzyme-linked immunosorbent assay

HA levels in conditioned media from 393P and 344SQ cells were measured using mouse HA enzyme-linked immunosorbent assay (ELISA) Kits (#DHYAL0; R&D Systems) according to the manufacturer's protocol.

Fluorescent cellular imaging

HA was stained using biotinylated HABP (#HKD-BC41; AMSBIO) according to the manufacturer's protocol. 4-MU (1 mg/mL; Sigma–Aldrich), a hyaluronan synthase inhibitor, was treated to cancer cells for 24 h as a control to deplete the HA matrix. Alexa Fluor-conjugated

phalloidin (Thermo Fisher Scientific) was used to stain the actin cytoskeleton. To assess HA matrix formation, we captured confocal images focused near the cell culture plate. The relative HA matrix area (HA staining area) was determined by dividing the total HA staining area by the phalloidin staining area, which represents the total cell body area. To visualize the HA cables connecting cancer cells, we captured confocal z-stack images, as these cables connect different focal planes between two cells. The extent of HA cable formation was quantified by dividing the total number of HA cables by the total number of cells in each image. RFP-tagged CD63 (pCT-CD63-RFP, System Biosciences) was used to visualize EVs. Cells were observed under a ZEISS confocal microscope (LSM800).

Mouse experiments

129/Sv mice were bred and housed in the Animal Facility at Ewha Medical Research Institute. BALB/c nude mice were purchased from Orient Bio. Syngeneic (129/Sv) mice were injected with 344SQ cells (5×10^5 or 1×10^6 cells per mouse), either subcutaneously in the right flank or orthotopically in the left lung. Additionally, 344SQ-mCherry cells (2×10^5 cells per mouse) were injected into the mice via the tail vein. Nude mice were orthotopically injected with HCC827 cells (1×10^6 cells per mouse). In the subcutaneous injection model, some mice developed severe metastatic disease, requiring euthanasia after 6 weeks. As a result, we chose a 6-week endpoint for assessing metastatic potential in this model. In both the orthotopic and tail vein injection models, 344SQ cells were introduced directly into the lungs, leading to faster tumor development compared to the subcutaneous model. Following orthotopic injection, we monitored systemic inflammation and infection by measuring complete blood count (CBC) parameters. The CBC results showed no significant changes in neutrophil levels before and after surgery. Although the white blood cell (WBC) count slightly increased post-surgery, it remained within the normal range (Supplemental Figure 3).

Due to the rapid progression of the disease, we euthanized the mice one week after orthotopic injection and ten days after tail vein injection to evaluate tumor development and colonization. After euthanasia, we analyzed the primary and colonized tumors. In the subcutaneous injection model, 344SQ cells primarily metastasized to the lungs. However, when the observation period extended beyond 6 weeks, 344SQ cells were also found to spread to multiple distant organs, including the intestines, heart, kidneys, adrenal glands, liver, spleen, paraaortic lymph nodes, and diaphragm, as previously documented (6-8). Therefore, our focus was on examining metastatic tumors in the lungs and other organs. However, since the studies were terminated before 6 weeks post-injection, only a few metastases were observed in the other organs. Mouse euthanasia was performed in accordance with IACUC guidelines, even before reaching the expected duration, when mice exhibited symptoms such as a significant reduction in body weight (typically more than 20% of baseline body weight), severe tumor burden (greater than 1.5 cm in diameter for subcutaneous injections), difficulty breathing or labored respiration, severe signs of pain or distress (e.g., hunched posture, reluctance to move, vocalizations, or excessive grooming), inability to eat or drink, severe dehydration (e.g., sunken eyes, skin tenting, or lethargy), paralysis or loss of mobility, severe wounds or ulcerations, and poor response to treatment.

Sincalide was administered intraperitoneally at a dosage of 2.5 mg/kg twice weekly. *In vivo* dosage considerations were based on *in vitro* data showing an IC50 of approximately 10 µM (equivalent to 11.43 mg/L), where sincalide exhibited no cytotoxicity and effectively inhibited cell migration and invasion. Using an estimated volume of distribution in mice of 0.2 L/kg and assuming 50% bioavailability for intraperitoneal administration, the calculated *in vivo* dosage was 4.6 mg/kg [(11.43 mg/L × 0.2 L/kg) / 0.5 = 4.6 mg/kg]. Considering previous research (1 mg/kg) (9) and our estimation, a dosage of 2.5 mg/kg was chosen, resulting in minimal side effects under this treatment regimen. A double dose of sincalide (5 mg/kg) was administered to

evaluate the maximum drug effect. Additionally, sincalide (2.5 mg/kg) was delivered via intravenous injection in the orthotopic model to expand the treatment approach.

Primary tumor volume was estimated using the equation: volume = 0.5 × length × width^2. To evaluate potential side effects, mice were administered with sincalide (2.5 mg/kg) twice weekly for six weeks. Blood and main organs (lung, liver, kidney, spleen, and heart) were then collected to examine any abnormalities resulting from sincalide treatment. CBC parameters were analyzed from the blood samples using an automated hematology analyzer XN-1000V (Sysmex). Additionally, plasma aspartate transaminase (AST), alanine transaminase (ALT), and blood urea nitrogen (BUN) levels were measured using ELISA kits (MyBioSource) to assess liver and kidney damage. Histological analysis of the main organs was conducted following H&E staining.

For the *in vivo* bioluminescence imaging, 344SQ cells transduced with pLenti-PalmGRET vector (a gift from Charles P. Lai; Addgene plasmid #158221) were subcutaneously injected into syngeneic mice. Prior to imaging, the mice were intraperitoneally injected with fluorofurimazine (2.5 mg/kg; Selleckchem). Bioluminescence was subsequently measured using the AniView 600pro imaging system (Biolight Biotechnology).

FDG-Position emission tomography/computed tomography (PET/CT) scanning

PET/CT scans were conducted after the mice were fasted for 12 hours, with unrestricted access to water. Anesthesia was induced using vaporized isoflurane (4% for induction and 2.5% for maintenance). To ensure proper hydration, 0.1 mL of sterile normal saline was administered subcutaneously. The scans were performed using PET/CT scanners (Biograph LOS, Siemens Healthcare and Discovery LS, GE Healthcare). Non-contrast CT images were captured from the skull base to the upper thigh, followed by PET imaging 60 minutes after intravenous injection of ^{18}F-FDG (8.65 ± 2.7 MBq) via the lateral tail vein. The standardized uptake value (SUV) was

determined using the formula: SUV = decay-corrected activity (kBq/mL of tissue volume) / body mass (g). Lesion SUVs were obtained by manually defining a volume of interest (VOI) around each lesion. Additionally, dynamic spiral CT imaging with contrast enhancement was performed using a Lightspeed Pro-16 multidetector CT scanner (GE Healthcare).

Immunohistochemistry

Formalin-fixed, paraffin-embedded tissue samples were prepared for staining. Sections (4 μm) were deparaffinized and rehydrated with xylene and ethanol. Antigen retrieval was conducted by heating the slides for 15 min in citrate buffer (pH 6.0) with 0.05% Tween-20. The following steps were performed using Rabbit Specific HRP/DAB (ABC) Detection IHC Kit (Abcam), according to the manufacturer's instructions. Sections were incubated overnight at 4 °C with primary antibodies: αSMA (1:100 dilution, #14-9760-82; Thermo Fisher Scientific), CD206 (1:50 dilution, #24595; Cell Signaling Technology), CK19 (1:200 dilution, #ab133496; Abcam), ZEB1 (1:100 dilution, #NBP1-05987; Novus Biologicals), and ITIH2 (1:100 dilution, #NBP2-31750; Novus Biologicals). Antibody binding was visualized using the VECTASTAIN Elite ABC-HRP Reagent (Vector Laboratories).

Drug-target interaction (DTI) analysis

A library of 2,459 FDA-approved drugs that passed clinical phase 4 trials was downloaded from the DRUGBANK database (10). To screen for drugs that may bind to the target protein ITIH2, the binding affinity between ITIH2 and each drug was predicted using DearDTI (11), a self-attention-based deep-learning model for drug-target binding affinity prediction. The molecular descriptors of the drugs were then calculated using RDkit (12). Based on the predicted binding affinities, molecular descriptors, and literature review, drug candidates that could interact with ITIH2 were manually selected for further analysis.

Surface Plasmon Resonance (SPR) analysis

The binding kinetics of ITIH2 to HA were analyzed using a Biacore 2000 instrument (GE Healthcare, Uppsala, Sweden) at iCLUEBIO. The NAD200M sensor chip was preconditioned with a flowing running buffer (10 mM HEPES, 150 mM NaCl, 0.005% Tween 20, pH 7.4). Biotinylated HA (100 μg/mL; Sigma-Aldrich) was injected over the activated sensor chip surface for 25 min at a flow rate of 5 μL/min. Subsequently, recombinant ITIH2 (100 nM; MyBioSource) and sincalide (132 nM to 8.47 μM) were injected over the HA-immobilized sensor chip surface at a flow rate of 10 μL/min, with real-time binding curves recorded. BIA evaluation software (GE Healthcare) was used for data analysis, and appropriate models were fitted to determine the equilibrium dissociation constants.

Supplemental References

1. Gibbons DL, Lin W, Creighton CJ, Rizvi ZH, Gregory PA, Goodall GJ, et al. Contextual extracellular cues promote tumor cell EMT and metastasis by regulating miR-200 family expression. *Genes Dev.* 2009;23(18):2140-51.
2. Bota-Rabassedas N, Banerjee P, Niu Y, Cao W, Luo J, Xi Y, et al. Contextual cues from cancer cells govern cancer-associated fibroblast heterogeneity. *Cell Rep.* 2021;35(3):109009.
3. Rauluseviciute I, Riudavets-Puig R, Blanc-Mathieu R, Castro-Mondragon JA, Ferenc K, Kumar V, et al. JASPAR 2024: 20th anniversary of the open-access database of transcription factor binding profiles. *Nucleic Acids Res.* 2024;52(D1):D174-d82.
4. Tsunoda T, and Takagi T. Estimating transcription factor bindability on DNA. *Bioinformatics.* 1999;15(7-8):622-30.
5. Kim EJ, Kim JS, Lee S, Cheon I, Kim SR, Ko YH, et al. ZEB1-regulated lnc-Nr2f1 promotes the migration and invasion of lung adenocarcinoma cells. *Cancer Lett.* 2022;533:215601.
6. Padhye A, Ungewiss C, Fradette JJ, Rodriguez BL, Albritton JL, Miller JS, et al. A novel ex vivo tumor system identifies Src-mediated invasion and metastasis in mesenchymal tumor cells in non-small cell lung cancer. *Sci Rep.* 2019;9(1):4819.
7. Ungewiss C, Rizvi ZH, Roybal JD, Peng DH, Gold KA, Shin DH, et al. The microRNA-200/Zeb1 axis regulates ECM-dependent β1-integrin/FAK signaling, cancer cell invasion and metastasis through CRKL. *Sci Rep.* 2016;6:18652.
8. Saintigny P, Massarelli E, Lin S, Ahn YH, Chen Y, Goswami S, et al. CXCR2 expression in tumor cells is a poor prognostic factor and promotes invasion and metastasis in lung adenocarcinoma. *Cancer Res.* 2013;73(2):571-82.

9. Iida N, Dzutsev A, Stewart CA, Smith L, Bouladoux N, Weingarten RA, et al. Commensal bacteria control cancer response to therapy by modulating the tumor microenvironment. *Science.* 2013;342(6161):967-70.
10. Wishart DS, Feunang YD, Guo AC, Lo EJ, Marcu A, Grant JR, et al. DrugBank 5.0: a major update to the DrugBank database for 2018. *Nucleic Acids Res.* 2018;46(D1):D1074-d82.
11. Shin B, Park S, Kang K, and Ho JC. Self-attention based molecule representation for predicting drug-target interaction. *Proc Mach Learn Res.* 2019;106:230-48.
12. RDKit: Open-Source Cheminformatics Software. https://www.rdkit.org. Accessed 07 Aug 2020.
13. Bartha Á, and Győrffy B. TNMplot.com: A Web Tool for the Comparison of Gene Expression in Normal, Tumor and Metastatic Tissues. *Int J Mol Sci.* 2021;22(5):2622.
14. Salcher S, Sturm G, Horvath L, Untergasser G, Kuempers C, Fotakis G, et al. High-resolution single-cell atlas reveals diversity and plasticity of tissue-resident neutrophils in non-small cell lung cancer. *Cancer Cell.* 2022;40(12):1503-20.e8.
15. Abdulla S, Aevermann B, Assis P, Badajoz S, Bell SM, Bezzi E, et al. CZ CELLxGENE Discover: a single-cell data platform for scalable exploration, analysis and modeling of aggregated data. *Nucleic Acids Res.* 2025;53(D1):D886-d900.
16. Chen EY, Tan CM, Kou Y, Duan Q, Wang Z, Meirelles GV, et al. Enrichr: interactive and collaborative HTML5 gene list enrichment analysis tool. *BMC Bioinformatics.* 2013;14:128.

Supplemental Table 1. qRT-PCR primers used in this study

Primers (Human)	Forward (5'-3')	Reverse (5'-3')
RPL32	ACAAAGCACATGCTGCCCAGTG	TTCCACGATGGCTTTGCGGTTC
ZEB1	GGCATACACCTACTCAACTACGG	TGGGCGGTGTAGAATCAGAGTC
ITIH2	GAAACCAGGACACGTCTTCCCA	CCACAATCTCTGAGCCTCCAAAG
HAS2	GTCATGTACACAGCCTTCAGAGC	ACAGATGAGGCTGGGTCAAGCA
ZEB2	AATGCACAGAGTGTGGCAAGGC	CTGCTGATGTGCGAACTGTAGG
VIM	AGGCAAAGCAGGAGTCCACTGA	ATCTGGCGTTCCAGGGACTCAT
SNAI1	CTGAGGCCAAGGATCTCCAG	TGCAGTATTTGCAGTTGAAGGC
SNAI2	ATCTGCGGCAAGGCGTTTTCCA	GAGCCCTCAGATTTGACCTGTC
TWIST1	GCCAGGTACATCGACTTCCTCT	TCCATCCTCCAGACCGAGAAGG
TWIST2	GCAAGATCCAGACGCTCAAGCT	ACACGGAGAAGGCGTAGCTGAG
FN1	ACAACACCGAGGTGACTGAGAC	GGACACAACGATGCTTCCTGAG
CDH1	GCCTCCTGAAAAGAGAGTGGAAG	TGGCAGTGTCTCTCCAAATCCG
CDH2	ATCTCGGGTCAGCTGTCGG	GGCTATCTGCTCGCGATCC
PARD6B	GTTTCAACGGCCAATCCACTGC	CTATGGTTGTCAGGACGCAATAC
INADL	ACAAGGCAGATTTGACGACCTGG	CTTTGAGCCACAACAGGAAGGTC
CLDN3	GCCTTCATCGGCAGCAACATCA	AGCGAGTCGTACACCTTGCACT
**Primers (Mouse)**	**Forward (5'-3')**	**Reverse (5'-3')**
Rpl32	ATCAGGCACCAGTCAGACCGAT	GTTGCTCCCATAACCGATGTTGG
Zeb1	ATTCAGCTACTGTGAGCCCTGC	CATTCTGGTCCTCCACAGTGGA
Zeb2	GCAGTGAGCATCGAAGAGTACC	GGCAAAAGCATCTGGAGTTCCAG
Cdh1	TACGGCGGTGGTGAGGACGA	GCCACACGGGGGAGACTTGC
Cdh2	CCTCCAGAGTTTACTGCCATGAC	CCACCACTGATTCTGTATGCCG
Crb3	CGGACCCTTTCACAAATAGCA	CGTTGGACTCATCACCTGGG
Snai1	CCCAAGGCCGTAGAGCTGA	GCTTTTGCCACTGTCCTCATC
Snai2	ATCCTCACCTCGGGAGCATA	TGCCGACGATGTCCATACAG
Twist1	TCGACTTCCTGTACCAGGTCCT	CCATCTTGGAGTCCAGCTCG
Vim	GCGTGCGGCTGCTTCAAGAC	ATGGCGTCGGCCAGCGAGAA
Cldn3	TCATCGTGGTGTCCATCCTGCT	AGAGCCGCCAACAGGAAAAGCA
Itih1	GTCCACTGAGAACAACGGATGG	GCAGTTCCACATCTGTCAGCAG
Itih2	ACCAGGACACATCCTCTCAGCT	CAGAACCTCCGAAGTAGTTGTGG
Itih3	CTCTTCAGCACCGATGTGACCA	ACCCTTCAGCAGCCCATCATTG
Itih4	CCTTTCCTGGAGAAGATGGCAC	CTGATGAGAGCAGTGGATTGGC
Itih5	AAGTGCTGCCTCTCCACAACAG	GCTTGTTGGACCACAGTAGGCT
Has1	GCTACTTCCACTGTGTGTCCTG	CTAAGCATTCGGTTGGTGAGGTG
Has2	CATCTGTGGAGATGGTGAAGGTC	AGCCATCCAGTATCTCACGCTG
Has3	CCTTGGCAACTCAGTGGACTAC	TGGACATCTCCTCCAACACCTC
Esrp1	GATGACAGCACAGTGGTGAGAG	CCTGAGCATTCAGGCAAAGTGC
Cd44	TCTTTATCCGGAGCACCTTG	GGTCACTCCACTGTCCTGGT
Acan	CAGGCTATGAGCAGTGTGATGC	GCTGCTGTCTTTGTCACCCACA
Bcan	CCGCTTCAATGTCTACTGCTTCC	TCTCCATCGCTTCCTGAGGCAG
Vcan	GGACCAAGTTCCACCCTGACAT	CTTCACTGCAAGGTTCCTCTTCT
Habp4	ATTCGGAAGCCAGAGTCCACAG	GGAAGACGTGAGACTCATCCTC
Cdc37	CAGAAGCTGGAAGACATGCGCA	CGAAGGTCTTGTGTTTCTGCTCC
Phbp	CCTCTGGAACTGAGTGCCACAT	GGTGTGGTCATAGAGTTGTCGG
Lyve1	ACCAGGTAGAGTCAGCGCAGAA	CAGGACACCTTTGCCATTCTTCC

Hmmr	GCAAAGCCAGTCACTTCTGCAG	AACTCAGCCAGGGCGAGCTTTA
Plod2	CATCCGAGAGTTCATTGCTCCAG	GCGCTGTCTTTCAGGTGAGTAC
Loxl2	TTCTGCCTGGAGGACACTGAGT	TCGGTGATGTCTATCCACTGGC
Fbln2	AGCCAGGCTATGTCCTCACAGA	GTAGAAGGAGCCCTTGGTGTTC
Hnf4a	CAGCAGTTAGCGCTCCGCCC	GGGGCTCCGCAAAGCCATCA

Supplemental Table 2. Antibodies used for Western blotting in this study

Antibody	company	catalog no.	titer
ITIH2	Novus Biologicals	NBP2-31750	1:500
ZEB1	Novus Biologicals	NBP1-05987	1:1,000
N-cadherin	Abclonal	A19083	1:1,000
E-cadherin	Abclonal	A20798	1:1,000
Vimentin	Abclonal	A19607	1:1,000
Phospho-ERK	Santa Cruz Biotechnology	sc-7383	1:1,000
ERK2	Santa Cruz Biotechnology	sc-154	1:1,000
CD44	Cell Signaling Technology	37259	1:1,000
α-tubulin	Novus Biologicals	NB100-690	1:10,000
β-actin	Bioworld	BS6007M	1:10,000

ZEB1 ChIP-seq in MCF7 breast cancer cells [GSE190095]

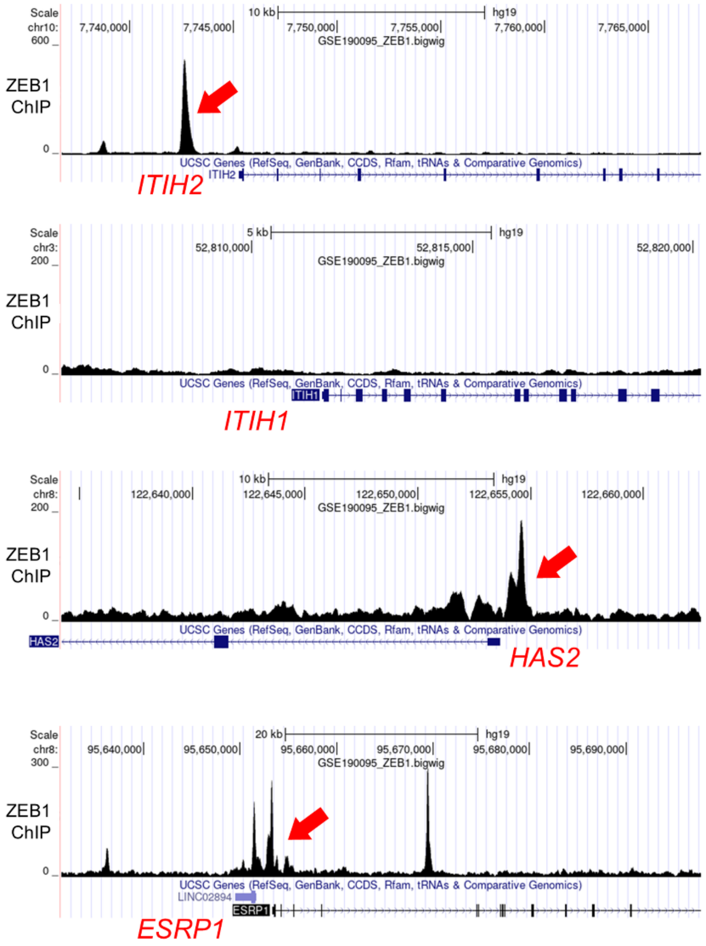

Supplemental Figure 1. ZEB1 binds to the promoter regions of *ITIH2*, *HAS2*, and *ESRP1.*
ZEB1 ChIP-sequencing data performed in MCF7 human breast cancer cells were obtained from the NCBI GEO (GSE190095). Red arrows indicate chromatin DNA fragments coimmunoprecipitated with ZEB1 aligned with each promoter region.

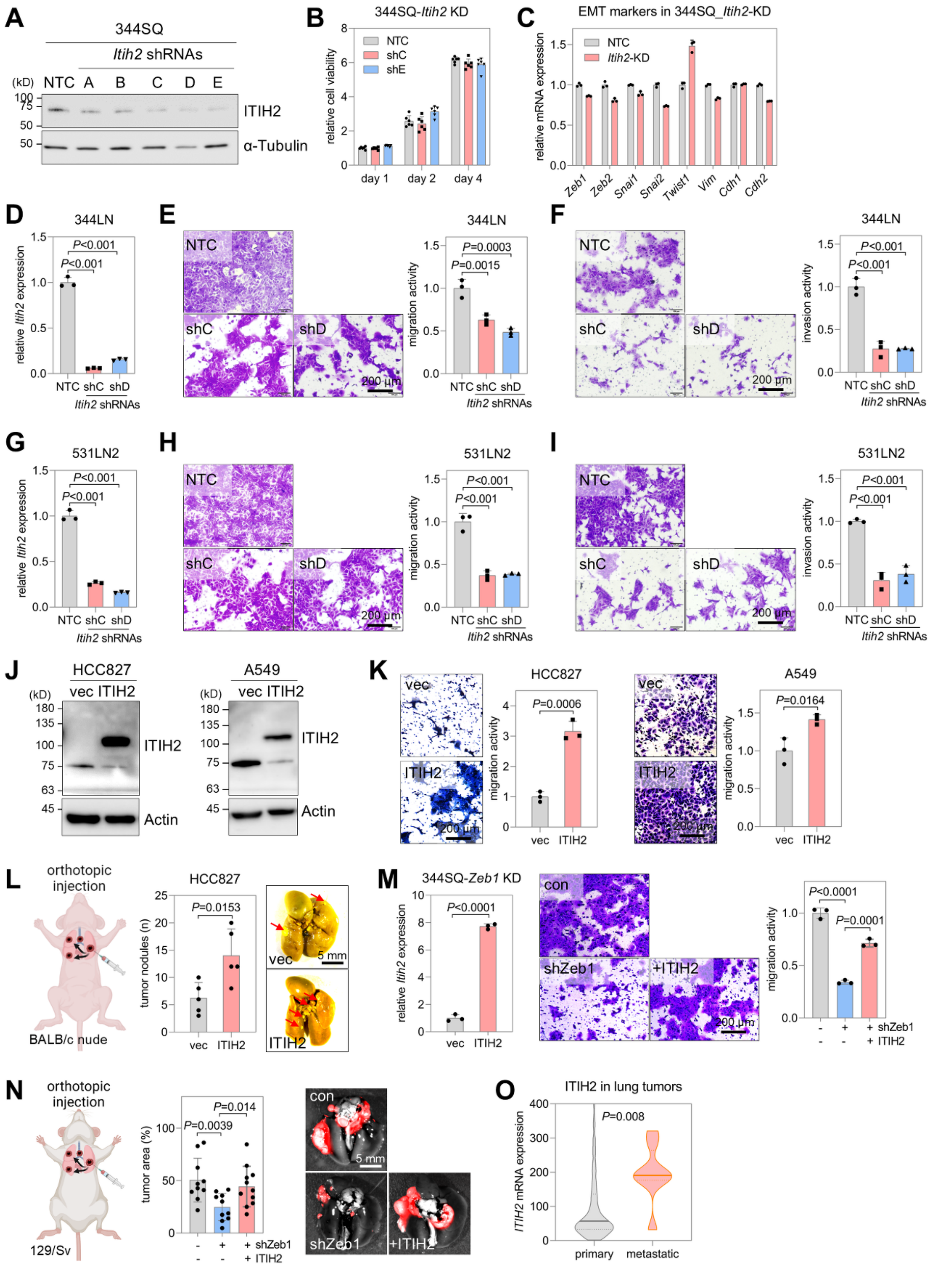

Supplemental Figure 2. ITIH2 promotes the migration and invasion of lung cancer cells.
(**A**) Western blot of ITIH2 in 344SQ cells transduced with *Itih2* shRNAs.
(**B**) WST-8 cell viability assay in 344SQ-ITIH2 KD cells. Mean ± SD (n = 6).
(**C**) qRT-PCR of EMT markers in 344SQ-ITIH2 KD cells.

(**D** and **G**) qRT-PCR of *Itih2* mRNA levels in 344LN (**D**) and 531LN2 cells (**G**) transduced with *Itih2* shRNAs. P, one-way ANOVA followed by Dunnett's multiple comparisons test.

(**E** and **H**) Boyden chamber migration assay in 344LN-ITIH2 KD (**E**) and 531LN2-ITIH2 KD cells (**H**). P, one-way ANOVA followed by Dunnett's multiple comparisons test.

(**F** and **I**) Boyden chamber invasion assay in 344LN-ITIH2 KD (**F**) and 531LN2-ITIH2 KD cells (**I**). P, one-way ANOVA followed by Dunnett's multiple comparisons test.

(**J**) Western blot of ITIH2 in HCC827 and A549 cells transfected with *ITIH2* cDNA.

(**K**) Boyden chamber migration assay in HCC827 and A549 cells overexpressing ITIH2

(**L**) Mouse orthotopic injection of HCC827 cells overexpressing ITIH2. Cells were injected into the left lung of BALB/c nude mice (n = 5). After 6 weeks, the total lung tumor nodules (red arrows) were measured at necropsy. P, two-tailed Student's t-test.

(**M**) Boyden chamber migration assay in 344SQ-ZEB1 KD transfected with *ITIH2* cDNA. qRT-PCR results of *Itih2* mRNA levels are shown in the left graph.

(**N**) Mouse orthotopic injection of 344SQ-ZEB1 KD transfected with *ITIH2* cDNA (n = 10 or 11). After a week of injection, the total lung tumor nodules were measured at necropsy.

(**O**) *ITIH2* mRNA levels in primary and metastatic lung tumors of human patients obtained from TNMplot (13). P, Mann–Whitney U test.

Data represent the mean ± SD from a single experiment with biological replicates (n = 3, unless otherwise specified) and are representative of at least three independent experiments.

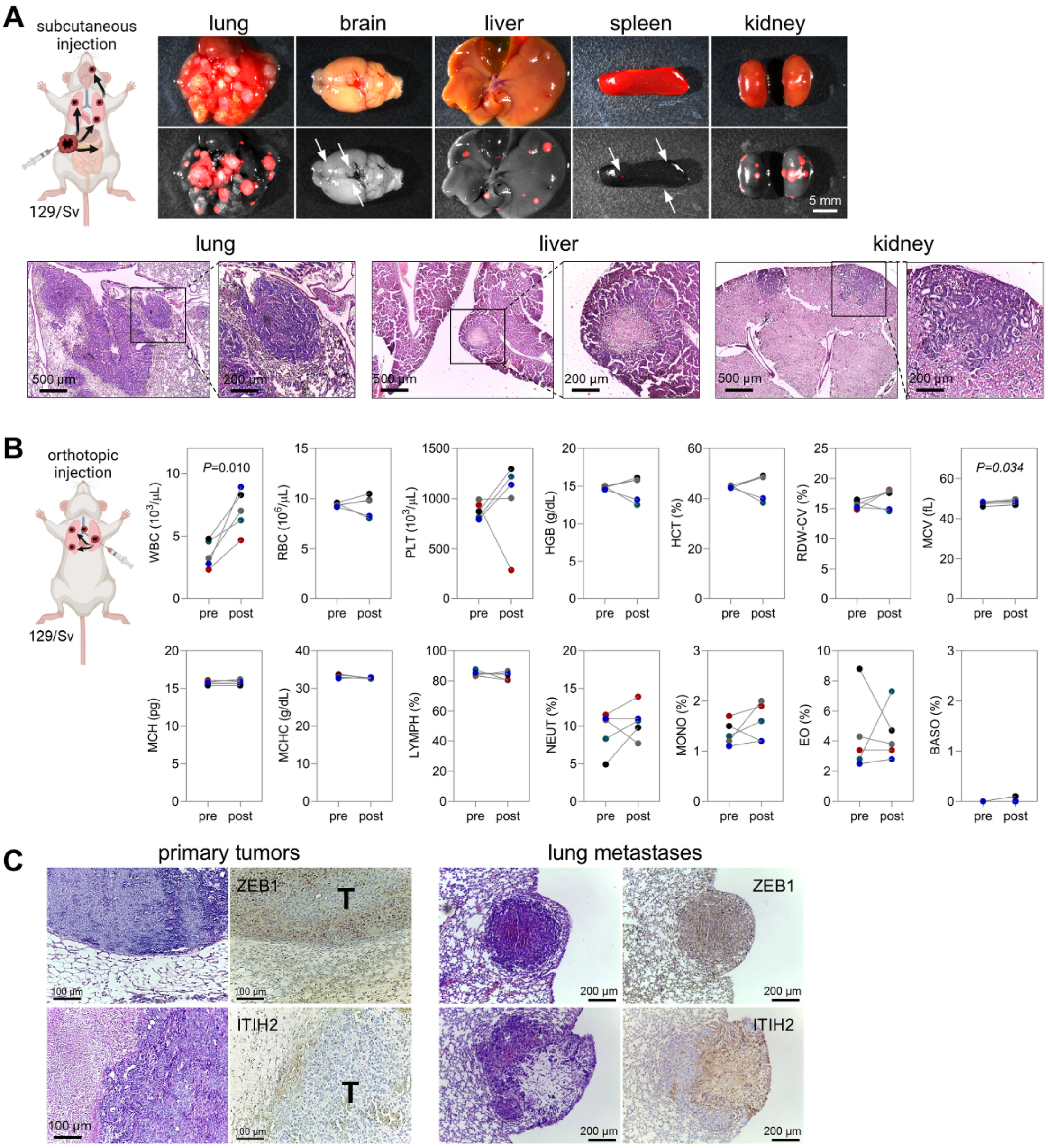

Supplemental Figure 3. Syngeneic mouse models used in this study.

(**A**) Mouse subcutaneous injection of 344SQ cells. 344SQ cells (5 × 10^5 cells per mouse) labeled with mCherry (red) were subcutaneously injected into the right flank. After 8 weeks, metastatic tumor nodules (red, white arrows) on the lungs and other organs were observed under a fluorescence stereomicroscope. H&E staining results of the metastatic tumors are shown in the lower panel. 344SQ-ITIH2 KD cells did not metastasize to other organs.

(**B**) The orthotopic lung injection procedure does not cause significant systemic inflammation. Hematology analysis was conducted on blood samples from mice before the surgical procedure for the orthotopic lung injection (pre) and seven days after (post). Complete blood count parameters were analyzed using an automated hematology analyzer.

(**C**) Immunohistochemical analysis of ZEB1 and ITIH2 was performed on primary and metastatic tumors from mice subcutaneously injected with 344SQ cells. T, tumor.

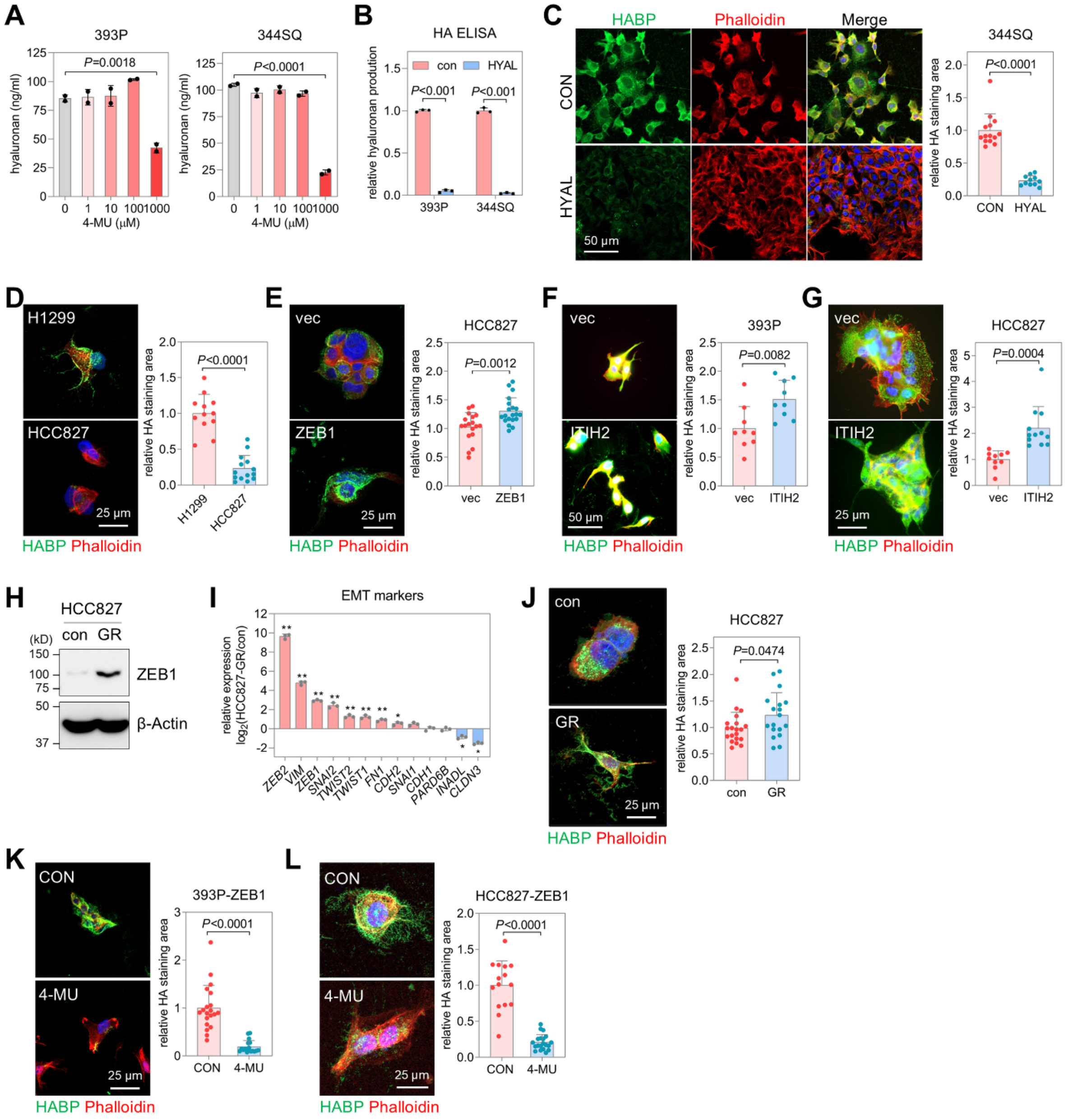

Supplemental Figure 4. ZEB1 and ITIH2 facilitate the formation of the hyaluronan (HA) matrix.

(**A**) ELISA analysis of HA levels in the culture media of 393P or 344SQ. P, one-way ANOVA followed by Dunnett's multiple comparisons test.

(**B**) ELISA analysis of HA levels in the culture media of 393P or 344SQ treated with hyaluronidase (HYAL) for 24 h. P, two-tailed Student's t-test.

(**C**) Confocal microscopy of 344SQ cells with HYAL treatment stained with HABP, phalloidin, and DAPI. Mean ± SD (CON, n = 13; HYAL, n = 10).

(**D**) Confocal microscopy of H1299 and HCC827 stained with HABP, phalloidin, and DAPI. Mean ± SD (H1299, n = 12; HCC827, n = 14).

(**E**) Confocal microscopy of HCC827-vec and HCC827-ZEB1 stained with HABP, phalloidin,

and DAPI. Mean ± SD (HCC827-vec, n = 18; HCC827-ZEB1, n = 20).

(**F** and **G**) Confocal microscopy of 393P-vec, 393P-ITIH2, HCC827-vec, and HCC827-ITIH2 stained with HABP, phalloidin, and DAPI. Mean ± SD (393P-vec, n = 9; 393P-ITIH2, n = 9; HCC827-vec, n = 10; HCC827-ITIH2, n = 12).

(**H**) Western blot of ZEB1 in HCC827 control (con) and gefitinib-resistant (GR) cells.

(**I**) qRT-PCR of EMT markers in HCC827-con and GR cells. $\log_2$-fold change (GR/con) values are presented. **P <0.01, *P <0.05, two-tailed Student's t-test.

(**J**) Confocal microscopy of HCC827-con and GR cells stained with HABP, phalloidin, and DAPI. Mean ± SD (HCC827-con, n = 20; HCC827-GR, n = 18).

(**K** and **L**) Confocal microscopy of 393P-ZEB1 and HCC827-ZEB1 with 4-MU treatment stained with HABP, phalloidin, and DAPI. Mean ± SD (393P-ZEB1+CON, n = 20; 393P-ZEB1+4-MU, n = 17; HCC827-ZEB1+CON, n = 15; HCC827-ZEB1+4-MU, n = 20).

Data represent the mean ± SD from a single experiment with biological replicates (n = 3, unless otherwise specified) and are representative of at least three independent experiments.

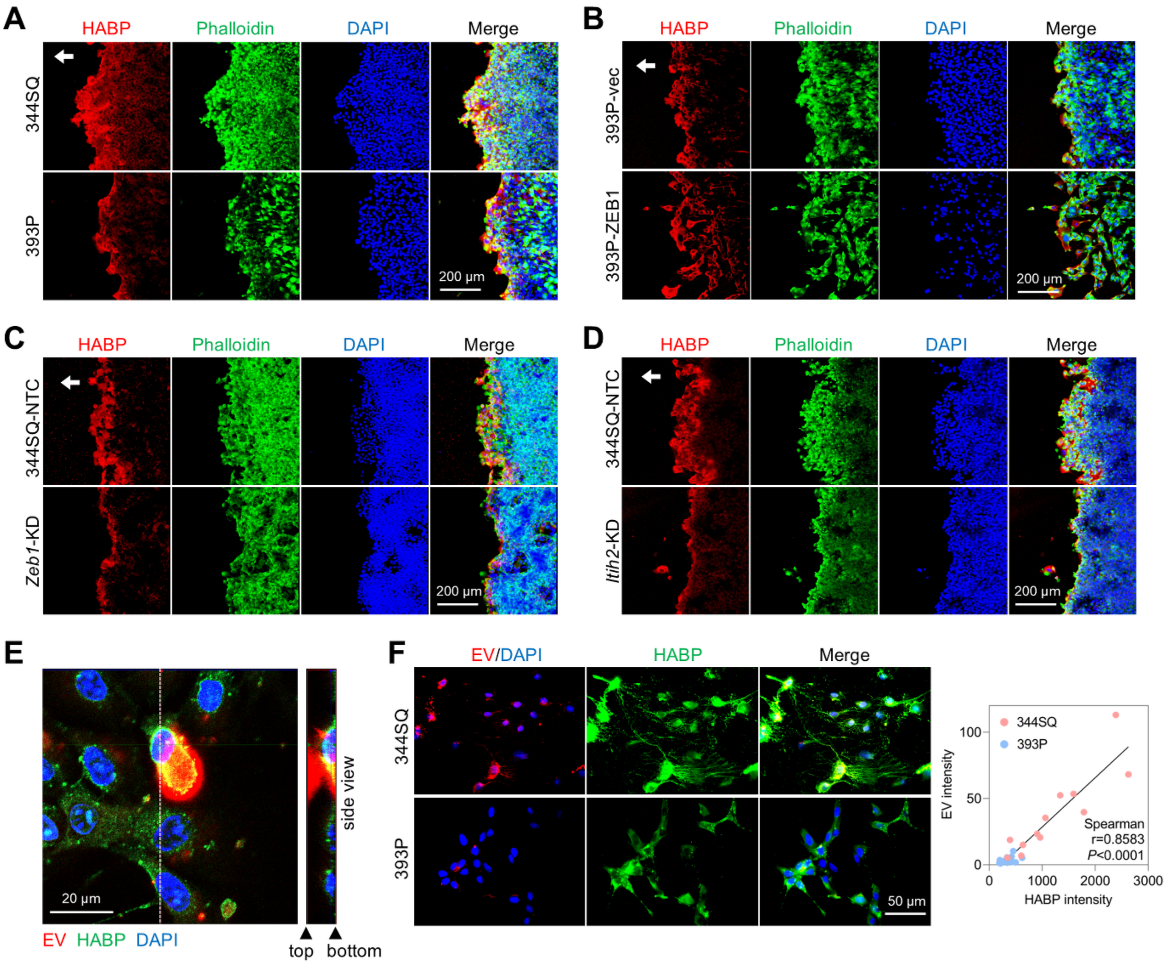

Supplemental Figure 5. ZEB1 and ITIH2 facilitate HA matrix formation.

(**A–D**) Confocal microscopy of 393P, 344SQ (A), 393P-vec, 393P-ZEB1 (B), 344SQ-NTC, 344SQ-ZEB1-KD (C), 344SQ-NTC, and 344SQ-ITIH2-KD (D) stained with HABP and streptavidin-Alexa594 (red), phalloidin (green), and DAPI (blue) in the two-well migration assay. White arrows indicate the direction of cell migration.

(**E**) Confocal z-stack images of 344SQ cells stained with HABP and streptavidin-Alexa488 (green), and DAPI (blue). EVs were isolated from 344SQ cells transfected with RFP-tagged CD63 and treated to 344SQ cells for 24 h. A side view of the confocal image is presented on the right. The same image in Figure 4K is shown again on the left.

(**F**) Confocal images of 393P and 344SQ cells stained with HABP and streptavidin-Alexa488 (green), and DAPI (blue). EVs expressing RFP-tagged CD63 were treated to cancer cells for 24 h. Staining intensity of EVs and HABP was quantified. Spearman's correlation r- and P-values are indicated.

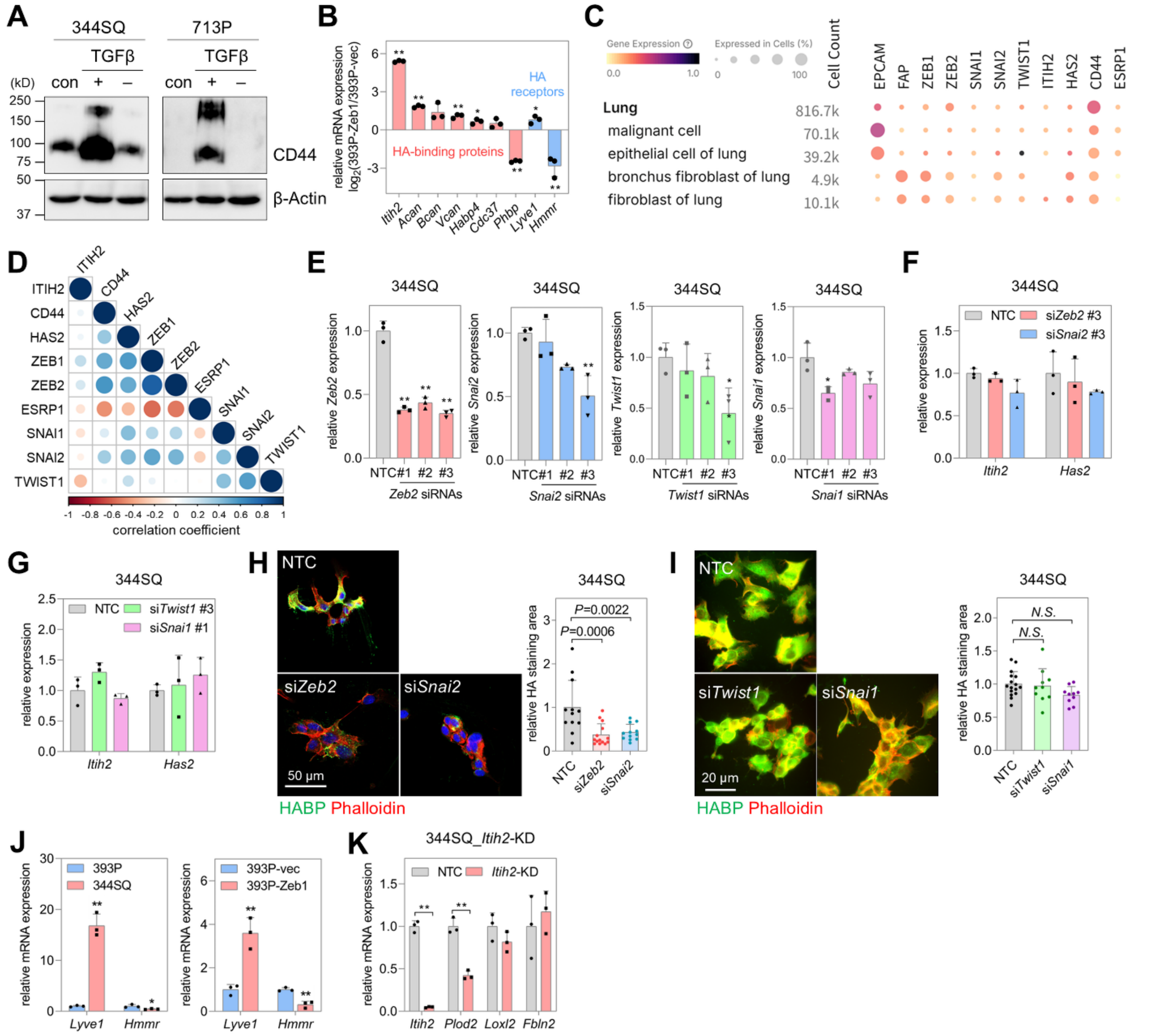

Supplemental Figure 6. ZEB1, but not other EMT-inducing transcription factors, regulates the HA network.

(**A**) Western blot of CD44 in 344SQ and 713P treated with TGFβ. The cells were treated with TGFβ (5 ng/mL) for a week, followed by an additional week of culture without TGFβ.

(**B**) qRT-PCR of HA-binding proteins and receptors in 393P-vec and 393P-ZEB1. **P <0.01, *P <0.05, two-tailed Student's t-test.

(**C**) Gene expression information from lung epithelial cells and fibroblasts in LUAD patients. The data was extracted from a single-cell transcriptomic dataset from 19 studies encompassing 309 LUAD patients (14) and analyzed using CZ CELLxGENE Discover (15). HA network-related genes (*ITIH2*, *HAS2*, *CD44*, and *ESRP1*) and EMT-transcription factors (*ZEB1*, *ZEB2*, *SNAI1*, *SNAI2*, and *TWIST1*) are expressed in both epithelial cancer cells (characterized by *EPCAM*) and fibroblasts (characterized by *FAP*), with EMT-transcription factors showing higher expression in fibroblasts.

(**D**) Correlation matrix between the HA network-related genes and EMT transcription factors in the TCGA-LUAD dataset.

(**E–G**) qRT-PCR of *Zeb2, Snai2, Twist1, Snai1, Itih2,* and *Has2* mRNA in 344SQ transfected with the respective siRNAs. **P <0.01, *P<0.05, one-way ANOVA followed by Dunnett's multiple comparisons test.

(**H** and **I**) Confocal microscopy of 344SQ transfected with the respective siRNAs, followed by staining with HABP and phalloidin. Mean ± SD (344SQ-NTC, n = 12; si*Zeb2*, n = 14; si*Snai2*, n = 12; NTC, n = 16; si*Twist1*, n = 10; si*Snai1*, n = 10). P, one-way ANOVA followed by Dunnett's multiple comparisons test. N.S., not significant.

(**J**) qRT-PCR of *Lyve1* and *Hmmr* mRNA in 393P, 344SQ, 393P-vec, and 393P-ZEB1.

(**K**) qRT-PCR of *Itih2*, *Plod2*, *Loxl2*, and *Fbln2* mRNA in 344SQ-ITIH2-KD cells.
Data represent the mean ± SD from a single experiment with biological replicates (n = 3, unless otherwise specified) and are representative of at least three independent experiments.

A

Name	CAS number	predicted Kd (nM)	predicted IC50 (nM)
Grazoprevir	1350514-68-9	0.226	0.39
Somatostatin	38916-34-6	2.237	1.734
Cangrelor	163706-06-7	1498.844	2.011
Voxilaprevir	1535212-07-7	1.499	2.595
Sincalide	25126-32-3	1.719	3.844
Asunaprevir	630420-16-5	22.756	3.901
Dinoprostone	363-24-6	383.448	5.307
Caspofungin	162808-62-0	25.201	6.408
Afamelanotide	75921-69-6	1.905	6.436

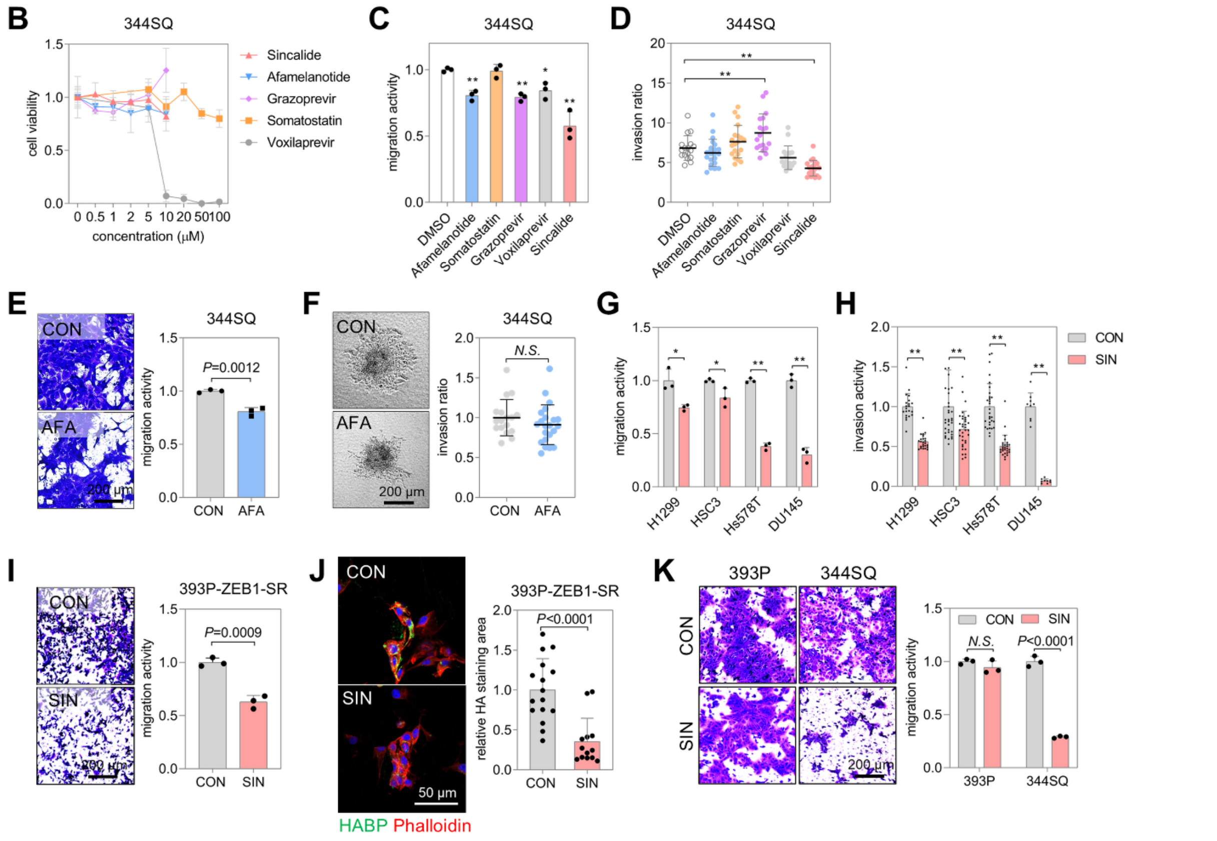

Supplemental Figure 7. ITIH2 inhibitor candidates inhibit the migration and invasion of cancer cells.

(**A**) Candidate inhibitors predicted through the MT-DTI algorithm.

(**B**) Cell viability of 344SQ treated with the ITIH2 inhibitor candidates.

(**C**) Boyden chamber migration assay of 344SQ treated with the candidates.

(**D**) Spheroid invasion assay of 344SQ treated with the candidates. Mean ± SD (DMSO, n = 17; afamelanotide, n = 22; somatostatin, n = 20; grazoprevir, n = 18; voxilaprevir, n = 17; sincalide, n = 21). **P <0.01, one-way ANOVA followed by Dunnett's multiple comparisons test.

(**E**) Migration assay of 344SQ treated with afamelanotide (AFA, 10 μM).

(**F**) Invasion assay of 344SQ treated with afamelanotide. Mean ± SD (CON, n = 19; AFA, n = 21). N.S., not significant.

(**G**) Migration assay of H1299 (lung), HSC3 (head and neck), Hs578T (breast), and DU145

(prostate cancer) cells treated with sincalide (SIN, 10 µM). **P <0.01, *P <0.05.

(**H**) Invasion assay of cancer cells treated with SIN. Mean ± SD (H1299_CON, n = 21; H1299_SIN, n = 24; HSC3_CON, n = 35; HSC3_SIN, n = 35; Hs578T_CON, n = 30; Hs578T_SIN, n = 28; DU145_CON, n = 9; DU145_SIN, n = 9).

(**I**) Migration assay of 393P-ZEB1 pre-treated with sincalide for six weeks to test potential resistance. Cells were then seeded in upper inserts with or without sincalide.

(**J**) Confocal microscopy of 393P-ZEB1 pre-treated with sincalide for six weeks. Cells were then cultured with or without sincalide for 24 h, followed by staining with HABP and phalloidin. Mean ± SD (CON, n = 16; SIN, n = 13).

(**K**) Migration assay of 393P and 344SQ treated with sincalide.

Data represent the mean ± SD from a single experiment with biological replicates (n = 3, unless otherwise specified) and are representative of at least three independent experiments.

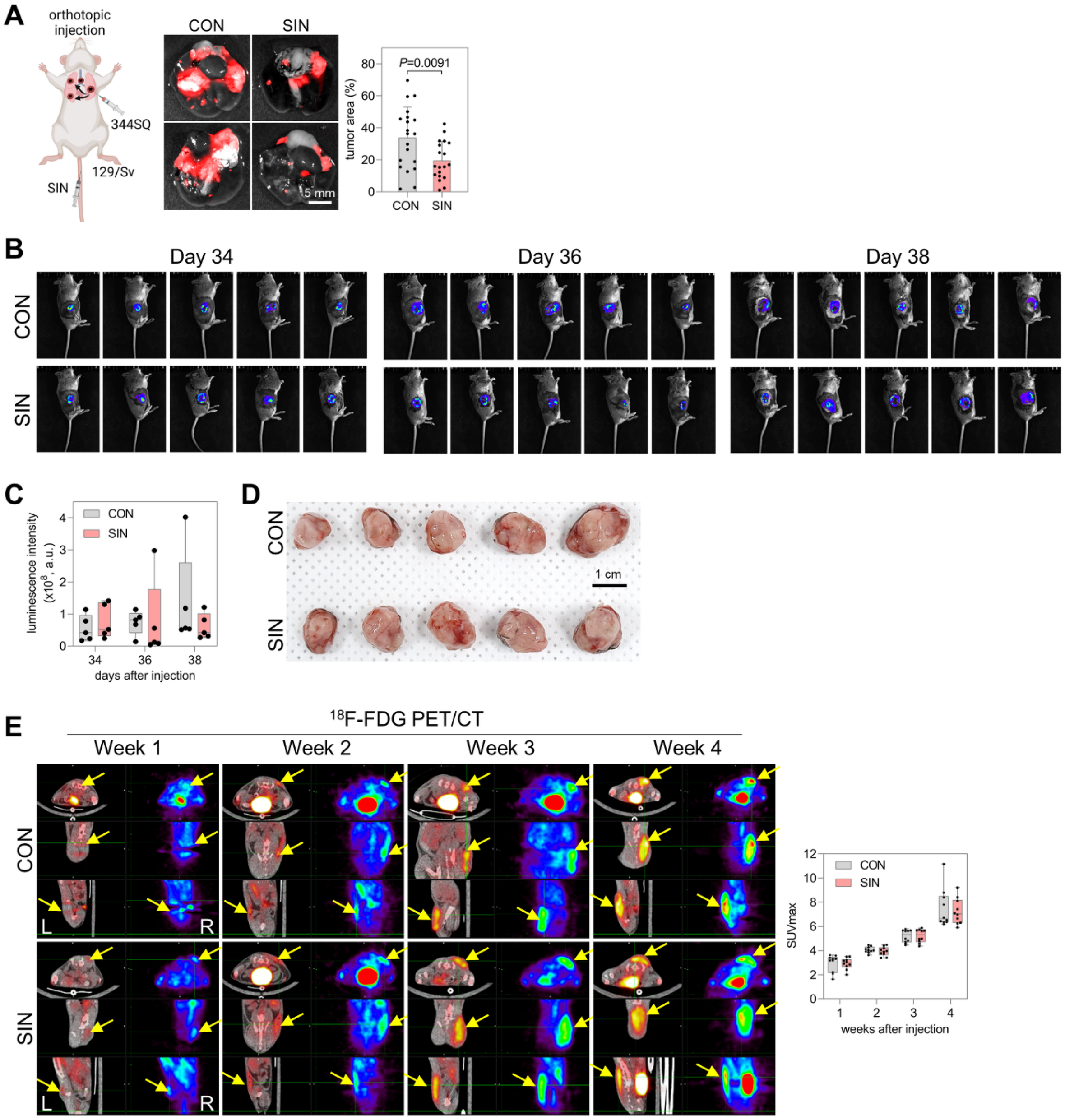

Supplemental Figure 8. Sincalide has little effect on primary tumor growth.

(**A**) 344SQ cells (mCherry) were orthotopically injected into the left lung (CON, n = 20; SIN, n = 19). Sincalide (2.5 mg/kg of body weight) was injected via the tail vein twice a week until mice were euthanized. After 7 days, tumor nodules on the lungs were measured at necropsy. P, two-tailed Student's t-test.

(**B-D**) In vivo bioluminescence imaging of primary tumors. 344SQ cells transduced with pLenti-PalmGRET were subcutaneously injected into 129/Sv syngeneic mice, with sincalide administered intraperitoneally twice a week. On Days 34, 36, and 38, the mice received an intraperitoneal injection of fluorofurimazine (2.5 mg/kg), and luminescence was subsequently measured using the AniView 600pro imaging system (B, C). On Day 42, the

mice were euthanized, and the tumors were excised for size comparison (D).

(**E**) PET/CT imaging of primary tumors. Tumor formation was monitored in mice following subcutaneous injection of 344SQ cells. PET/CT scans were performed every seven days for a total of 30 days, starting on Day 7 post-implantation. Sincalide (2.5 mg/kg body weight) was administered intraperitoneally twice a week until the mice were euthanized.

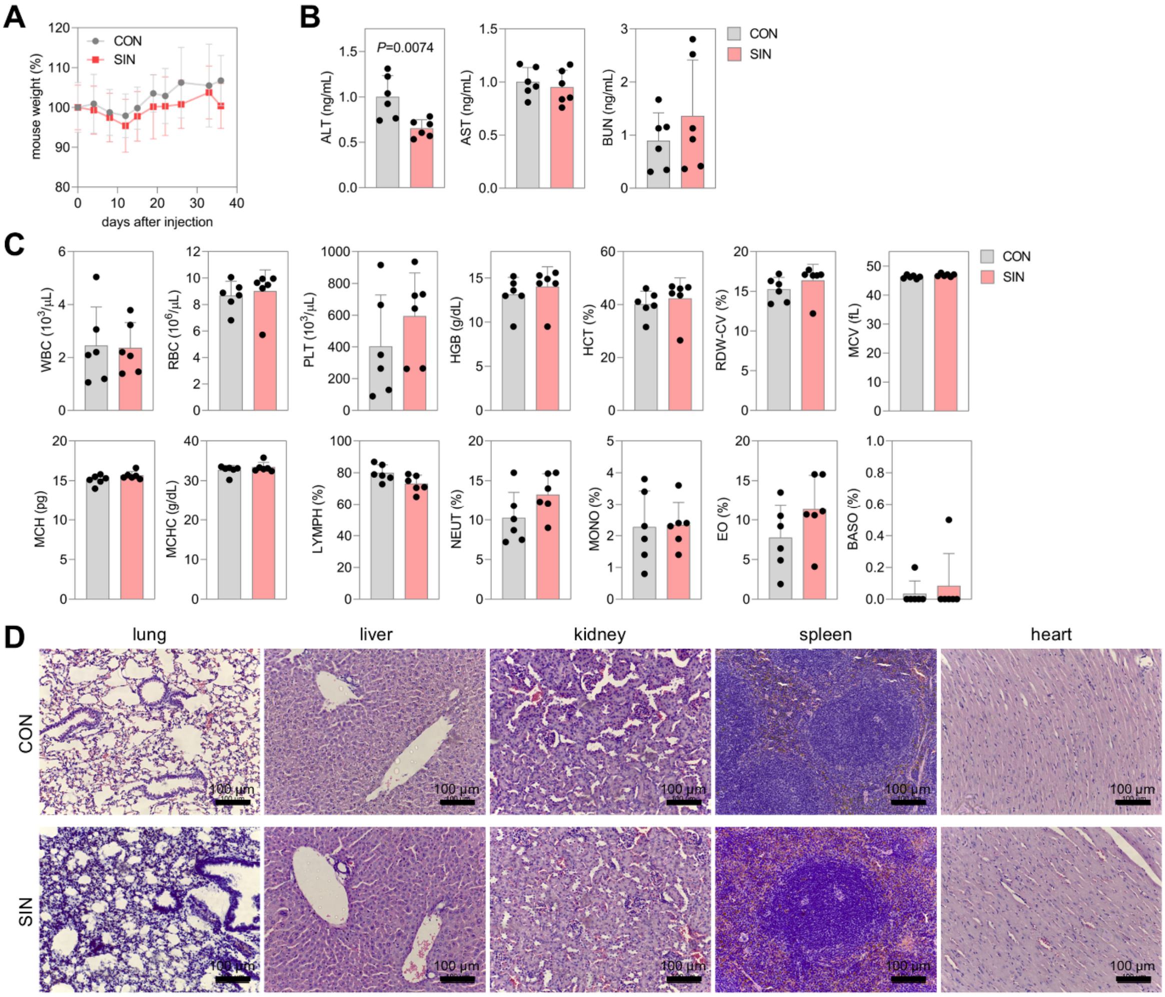

Supplemental Figure 9. Sincalide has no adverse effects in mice.
(**A**) Changes in mouse weight during the sincalide treatment.
(**B**) ELISA to test liver and kidney toxicity. After six weeks of sincalide treatment, mouse blood was collected, and plasma AST (aspartate aminotransferase), ALT (alanine transaminase), and BUN (blood urea nitrogen) levels were measured using the ELISA kits. Mean ± SD (n = 6). P, two-tailed Student's t-test.
(**C**) Hematology analysis of blood samples from mice treated with sincalide. Complete blood count parameters were analyzed using an automated hematology analyzer.
(**D**) H&E staining of tissues from mice treated with sincalide.

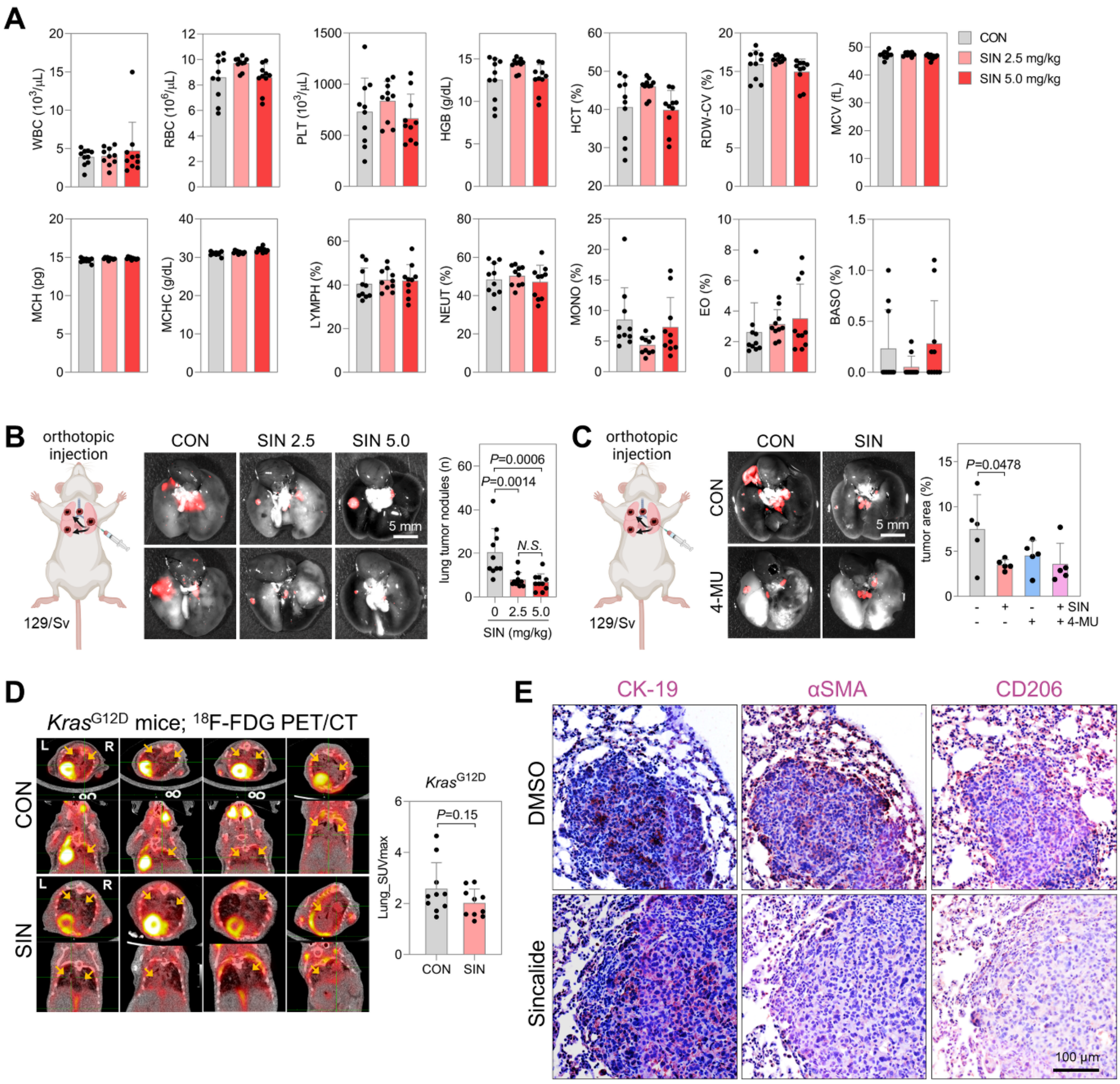

Supplemental Figure 10. Sincalide inhibits lung cancer progression.

(**A**) Hematology analysis of blood samples from mice intraperitoneally treated with sincalide (2.5 or 5.0 mg/kg of body weight). Complete blood count parameters were analyzed using an automated hematology analyzer.

(**B**) 344SQ cells (mCherry) were orthotopically injected into the left lung (n = 10 per group). Sincalide (2.5 or 5.0 mg/kg of body weight) was injected intraperitoneally twice a week until mice were euthanized. After 7 days, the number of lung tumor nodules was measured at necropsy. P, one-way ANOVA followed by Tukey's multiple comparisons test. N.S., not significant.

(**C**) 344SQ cells (mCherry) were orthotopically injected into the left lung (n = 5 per group). Sincalide (2.5 mg/kg of body weight) and 4-MU (20 mg/kg of body weight) were injected intraperitoneally twice a week until mice were euthanized. After 7 days, lung tumor nodules

were measured at necropsy. P, one-way ANOVA followed by Dunnett's multiple comparisons test.

(**D**) Sincalide treatment in a spontaneous lung cancer mouse model. After 8 weeks of Adeno-Cre injection in *Kras*$^{LSL\text{-}G12D}$ mice, sincalide (2.5 mg/kg of body weight) was administered intraperitoneally twice a week until the mice were euthanized. Lung tumor growth was assessed using ^{18}F-FDG PET/CT after 4 weeks of treatment. P, two-tailed Student's t-test.

(**E**) Immunohistochemical analysis of CK19 (a marker for epithelial cancer cells), αSMA (a marker for active CAFs), and CD206 (a marker for M2 macrophages) was performed on lung tumors from mice orthotopically injected with 344SQ cells and treated with sincalide.

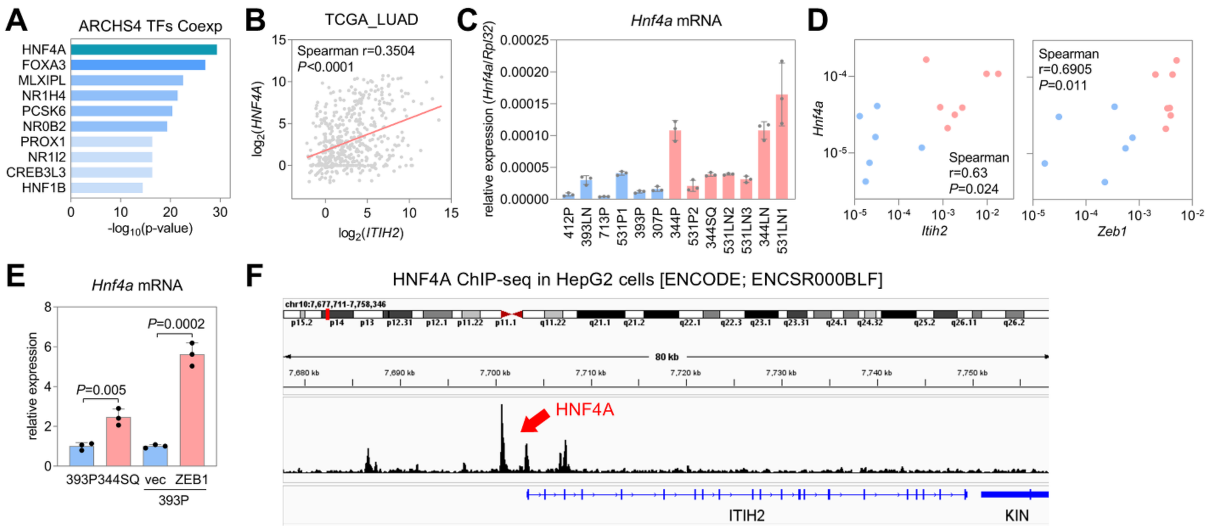

Supplemental Figure 11. HNF4A is a candidate transcription factor of ITIH2.

(**A**) ARCHS4 transcription factors co-expression analysis on 242 genes whose mRNA expression levels positively correlate (Spearman's r > 0.3, q < 1.0E-06) with those of *ITIH2* in TCGA-LUDA data. The enrichment analysis was performed using Enrichr (16).

(**B**) Scatter plot depicting the correlation between *HNF4A* and *ITIH2* mRNA levels in TCGA-LUAD data (n = 517).

(**C**) qRT-PCR analysis of *Hnf4a* mRNA in epithelial-like (blue) and mesenchymal-like (red) murine lung cancer cells.

(**D**) Scatter plots illustrating the correlation between *Zeb1, Hnf4a,* and *Itih2* mRNA levels in qRT-PCR data of murine lung cancer cells (n = 13).

(**E**) qRT-PCR of *Hnf4a* mRNA in 393P, 344SQ, 393P-vec, and 393P-ZEB1 cells. P, two-tailed Student's t-test.

(**F**) HNF4A ChIP-sequencing data performed in HepG2 human hepatocellular carcinoma cells were obtained from the ENCODE (ENCSR000BLF). Red arrows indicate chromatin DNA fragments coimmunoprecipitated with HNF4A aligned with the *ITIH2* promoter region. Data represent the mean ± SD from a single experiment with biological replicates (n = 3, unless otherwise specified) and are representative of at least three independent experiments.
