# Supplementary figures and images for: Hyaluronan network remodeling by ZEB1 and ITIH2 enhances the motility and invasiveness of cancer cells
Source: J Clin Invest. 2025 Apr 3;135(11):e180570. doi: 10.1172/JCI180570 (PMC12126249; doi:10.1172/JCI180570)

**Fig. 1A**

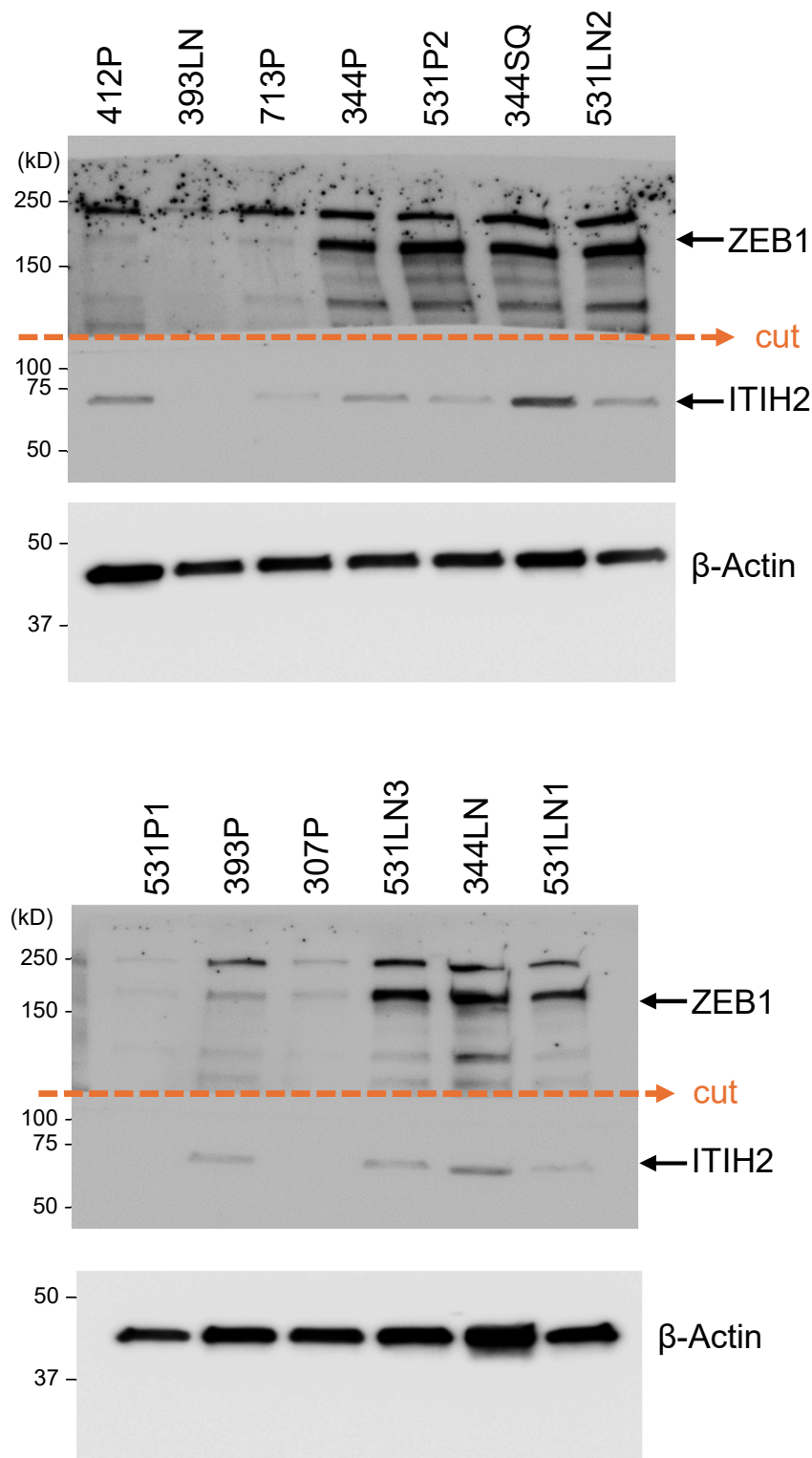

**Fig. 1G**

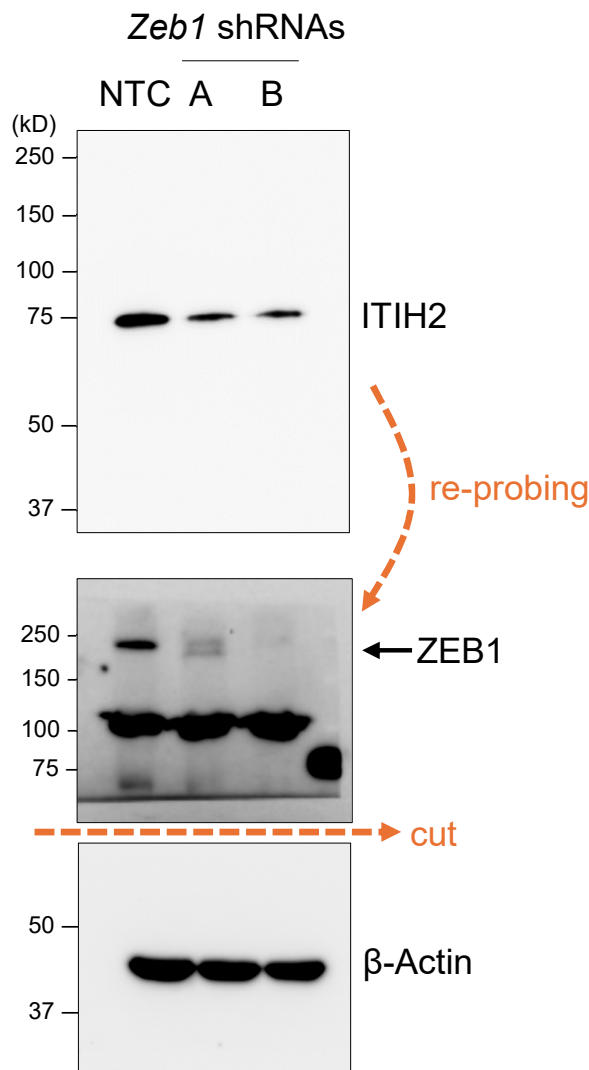

**Fig. 1I (ChIP – PCR)**

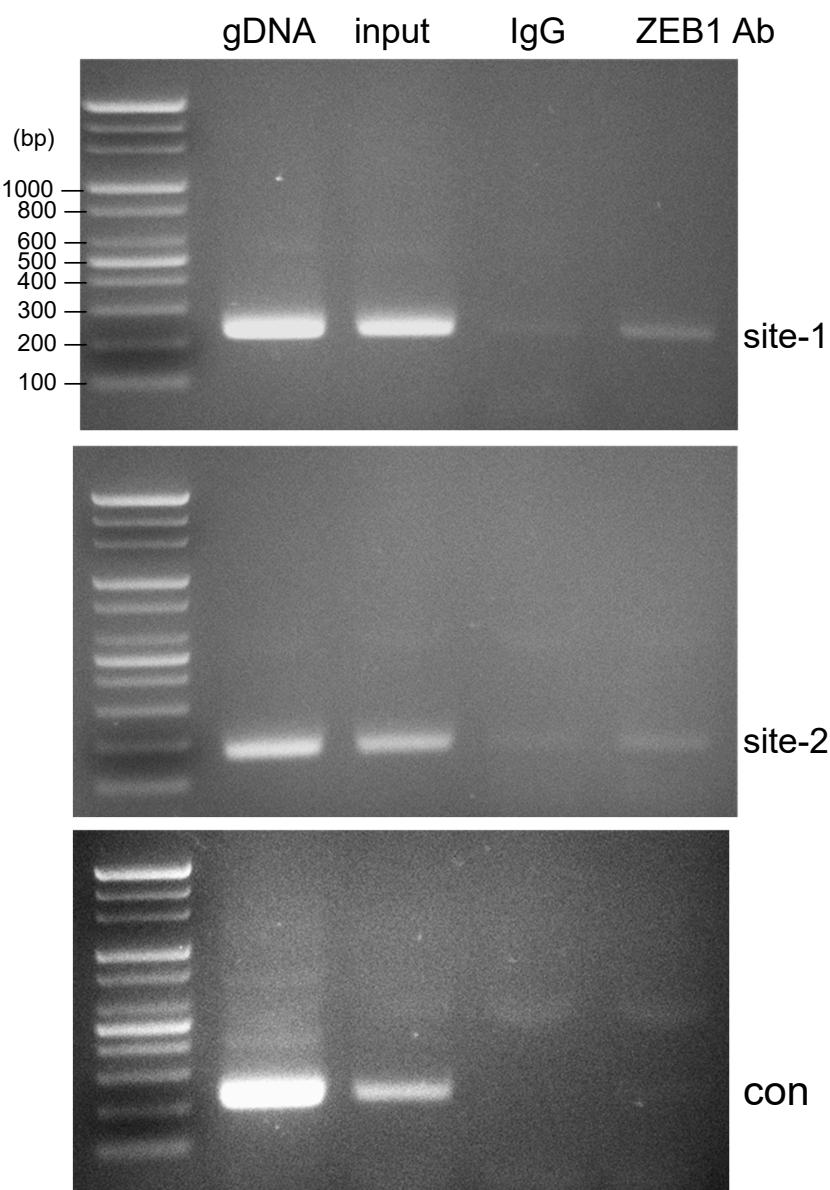

**Fig. 5D**

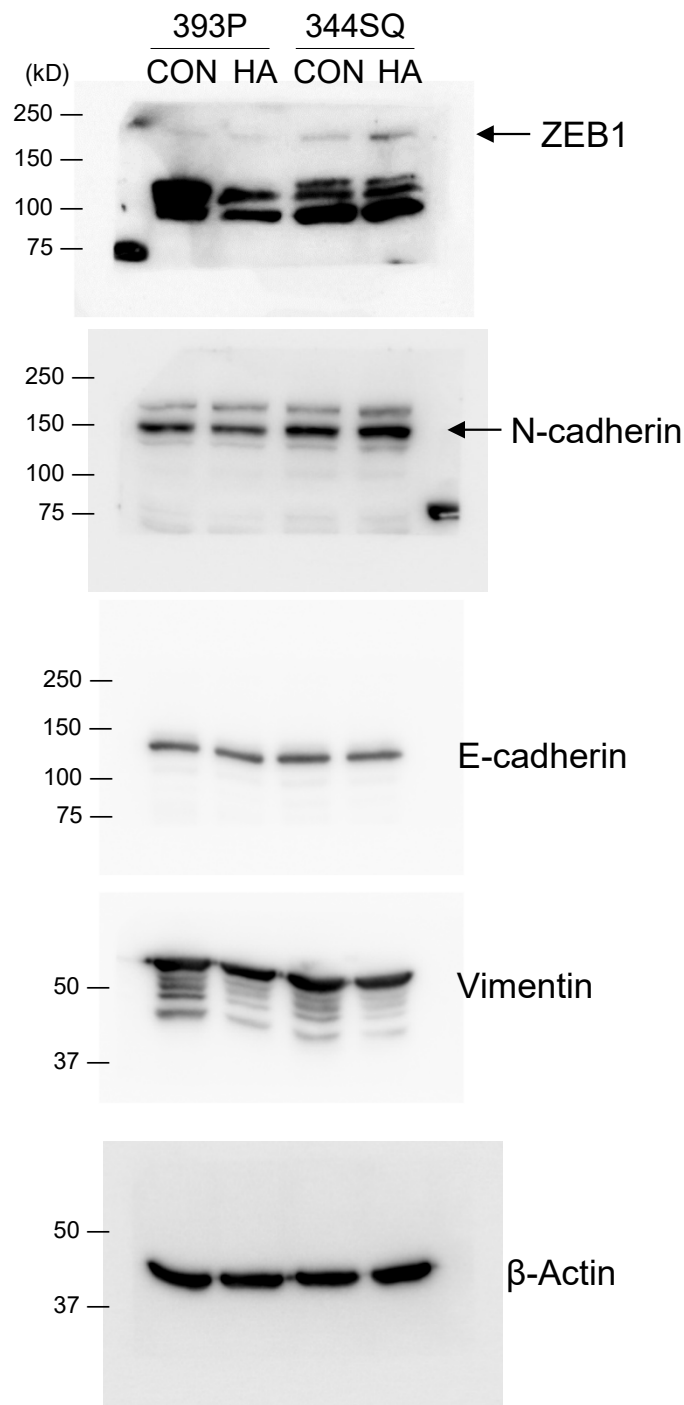

**Fig. 5G**

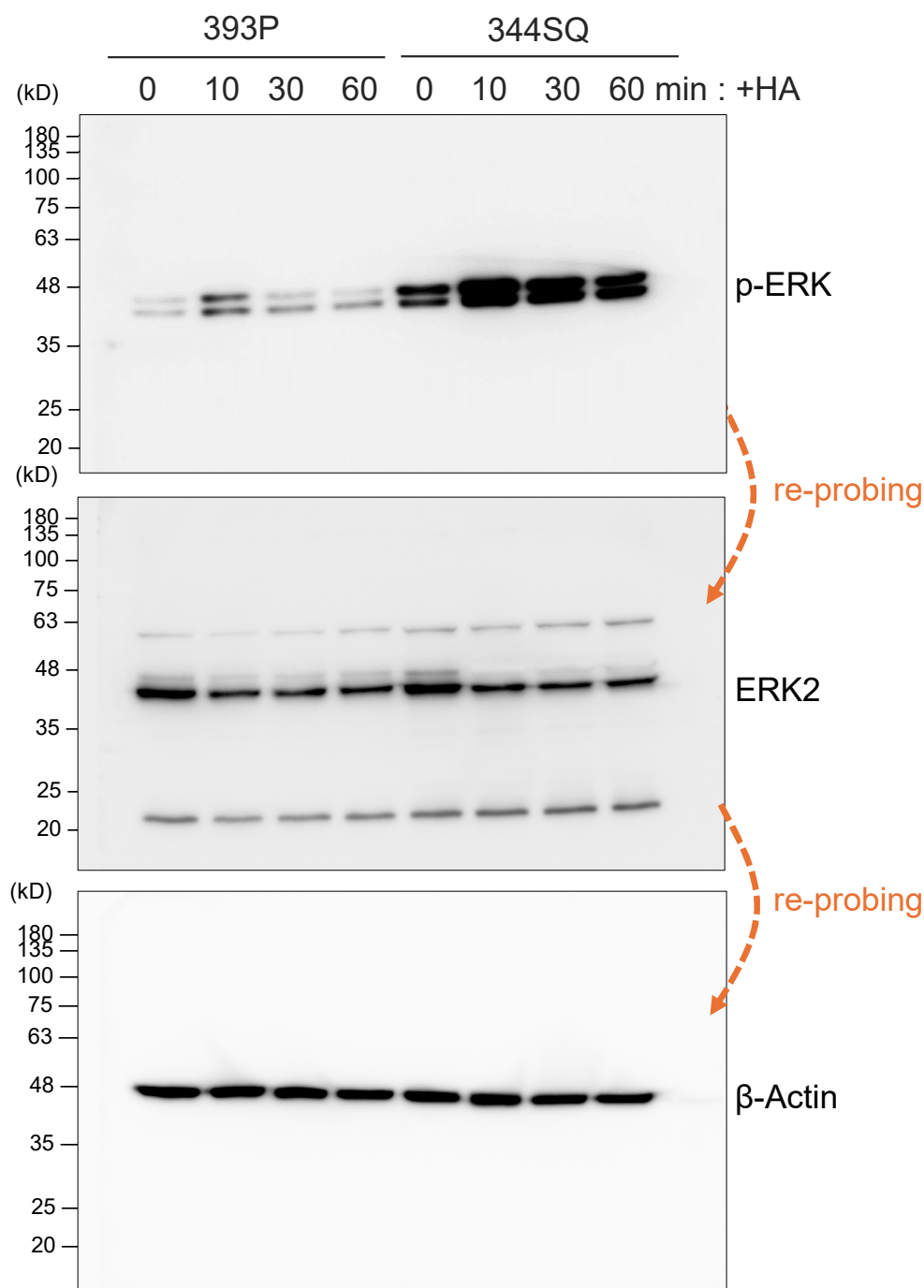

Fig. 7A

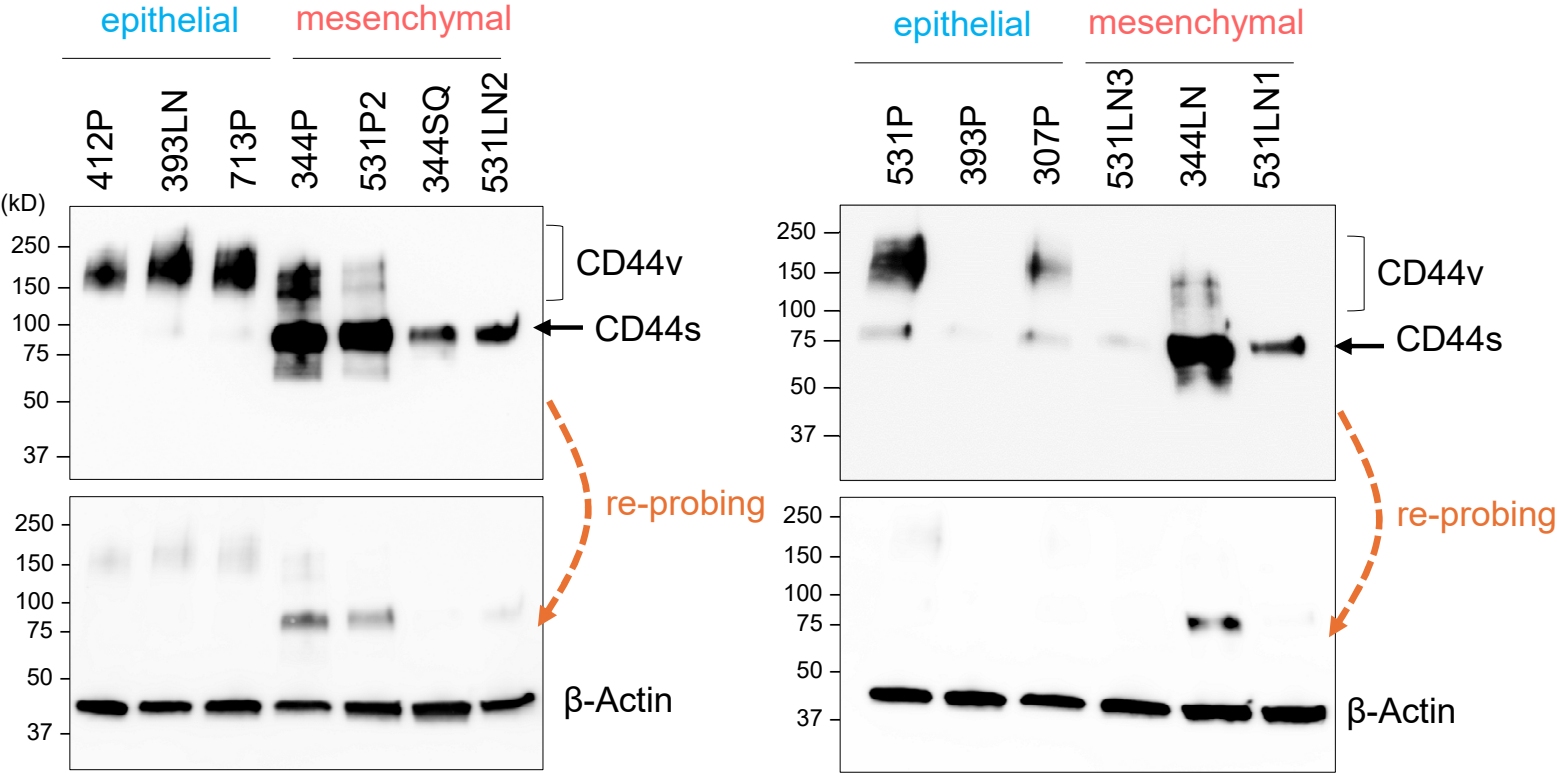

Fig. 7B (RT-PCR)

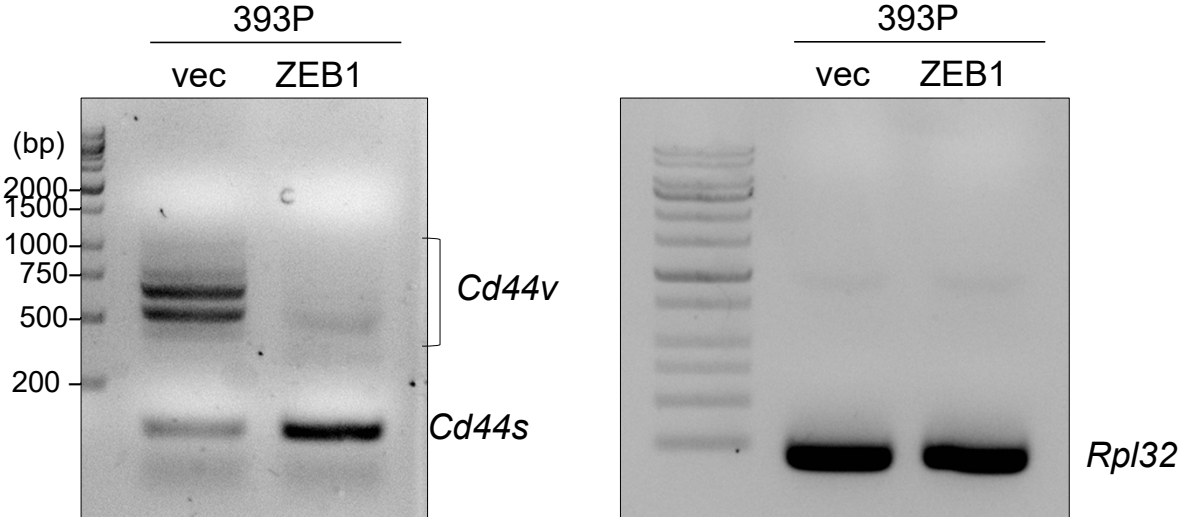

**Fig. 7C**

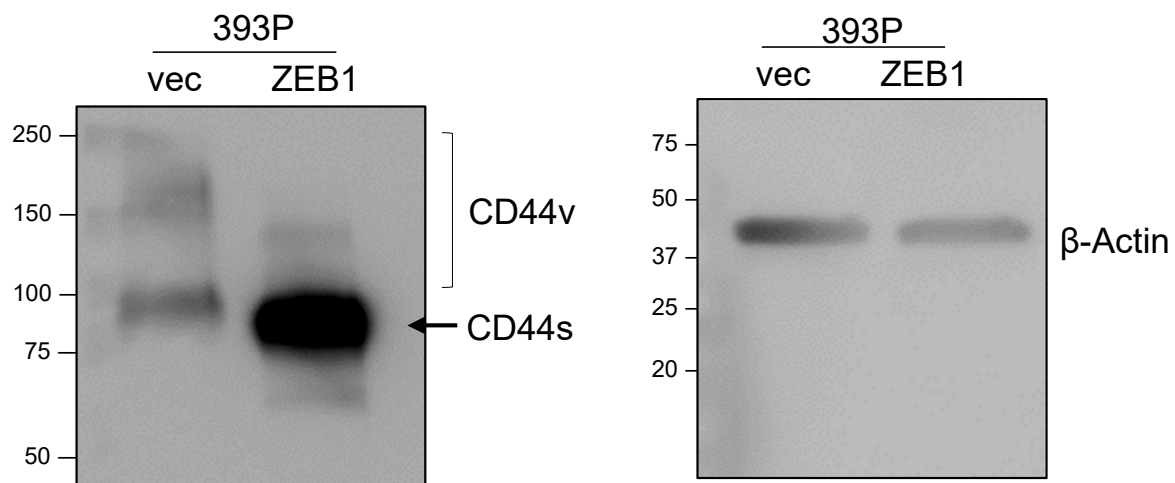

**Fig. 7D**

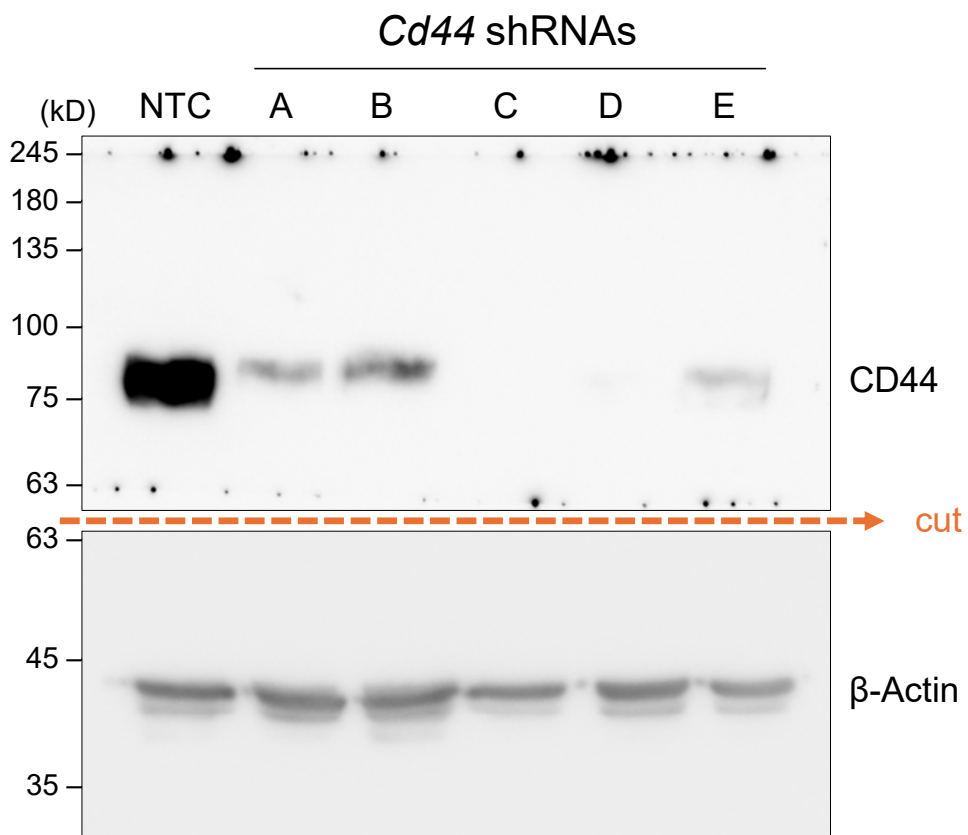

**S\_Fig. 2A**

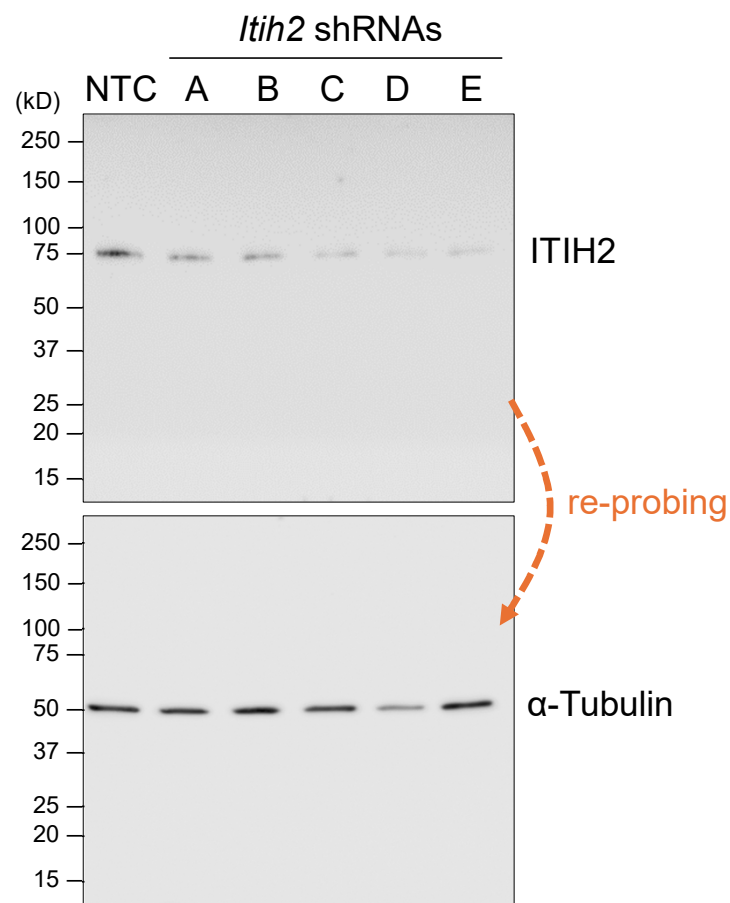

**S\_Fig. 2H**

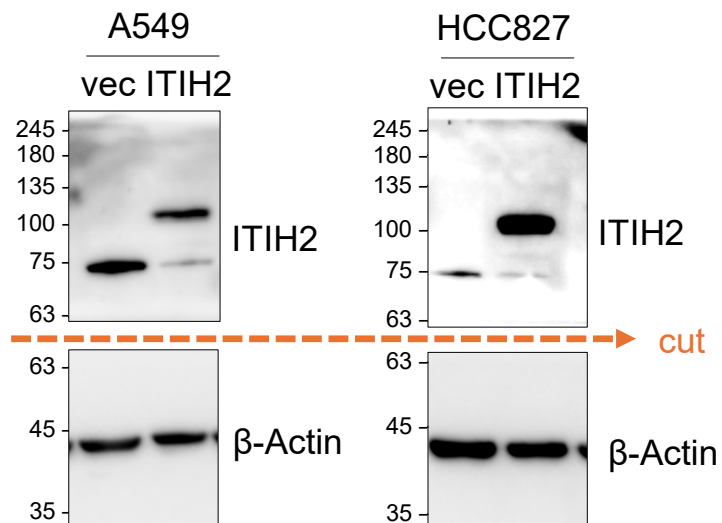

**S\_Fig. 3H**

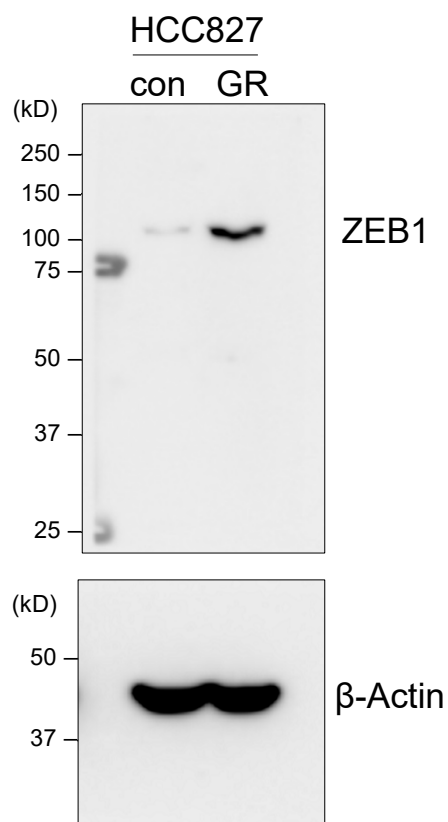

**S\_Fig. 5A**

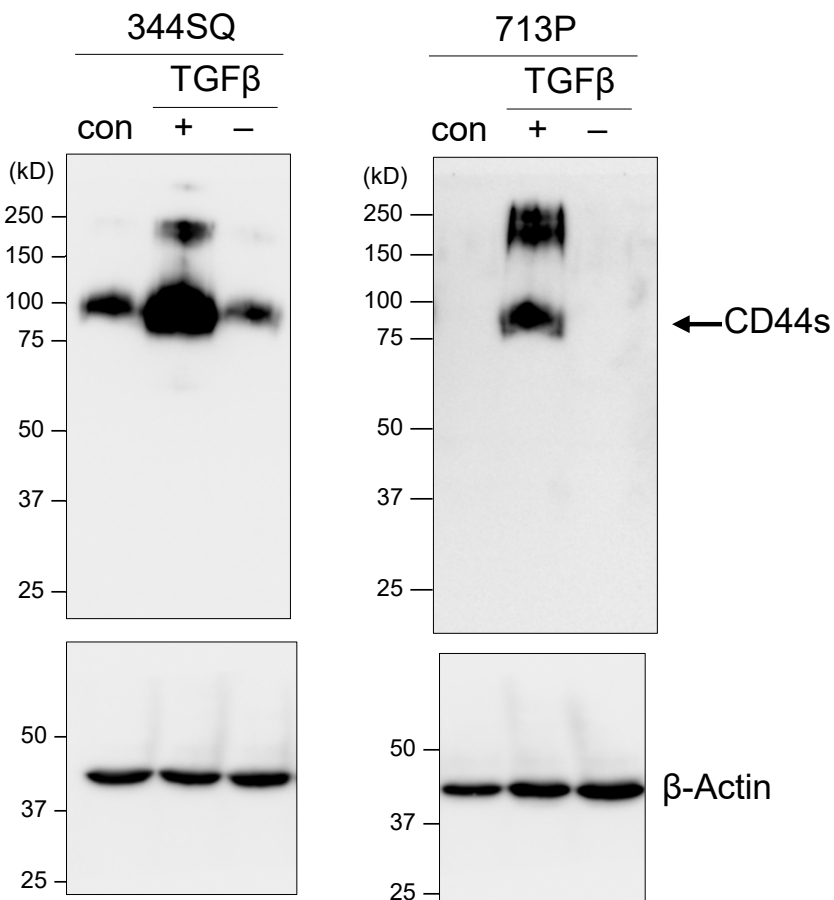

Supplement: Unedited blot and gel images [file jci-135-180570-s066.pdf]
